# PRDM16 Reduces Cellular Senescence by Upregulating GSTM1

**DOI:** 10.1002/advs.202501233

**Published:** 2025-09-14

**Authors:** Qian Yuan, Yuting Zhu, Ben Tang, Yaru Xie, Mingcun Hu, Hua Su, Youhua Liu, Chun Zhang

**Affiliations:** ^1^ Department of Nephrology Union Hospital Tongji Medical College Huazhong University of Science and Technology Wuhan 430022 China; ^2^ State Key Laboratory of Organ Failure Research National Clinical Research Center of Kidney Disease and Division of Nephrology Nanfang Hospital Southern Medical University Guangzhou 510515 China

**Keywords:** aging, glutathione‐S‐transferase, oxidative DNA damage, PRDM16, senescence

## Abstract

Cellular senescence is a hallmark of aging and the accumulation of senescent cells (SnCs) accelerates the aging process, contributing to aging‐related organ disorders. The PRDF1 and RIZ1 homology domain (PRDM) protein exhibits robust transcriptional regulatory activities and governs a wide range of biological processes. However, its roles in cellular senescence remain unclear. Here, this work demonstrates that PRDM16, a member of the PRDM protein family, decreases significantly in multiple organs of aged mice compared to young mice. Global *Prdm16* deletion contributes to cellular senescence in various organs, including the kidneys, heart, lungs, hippocampus, stomach, and gut, leading to accelerated aging‐related organ injury. Furthermore, tubular‐specific *Prdm16* deletion aggravates irradiation‐induced kidney aging and aging‐related kidney diseases in irradiated mice subjected to ischemia‐reperfusion surgery. Exogenous PRDM16 gene delivery by lentivirus effectively attenuates cellular senescence in vitro and in vivo. Mechanistically, PRDM16 improves glutathione metabolism and inhibits oxidative DNA damage, which is a driving force of senescence. Specifically, PRDM16 upregulates the transcription of glutathione S‐transferase mu 1 (GSTM1) by binding to its promoter region. Transfection with GSTM1 reverses PRDM16 deficiency‐induced cellular senescence and kidney aging. Collectively, these results provide a potential target for the investigation of anti‐aging therapies.

## Graphical abstract

Cellular senescence drives aging and aging‐related organ disorders, yet PRDM16's role remains unexplored. This work uncovers that PRDM16 decreases significantly in aged organs, while its loss accelerates cellular senescence and aging‐related organ injury. Crucially, restoring PRDM16 attenuates cellular senescence by activating GSTM1 and improving glutathione metabolism to mitigate oxidative DNA damage—revealing a promising anti‐aging target.

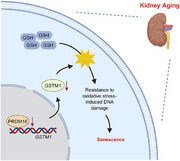

## 1 Introduction

With an increasing human lifespan, the burden of aging and aging‐related diseases has emerged as a global public health concern.^[^ ^1^ ^]^ Aging is a risk factor for various organ diseases. The incidences of kidney disease, cardiovascular disease, lung fibrosis, diabetes, and neurodegenerative disease have increased significantly over time.^[^ ^2^ ^]^

Cellular senescence is a hallmark of aging,^[^ ^3^ ^]^ and senescent cells (SnCs) progressively accumulate in multiple organs with aging. Accumulation of SnCs has been implicated in aging‐related disorders of various organs, including the kidneys, lungs, heart, brain, skin, stomach, and gut.^[^ ^4^ ^]^ The senescence‐associated secretory phenotype (SASP) of SnCs contributes to chronic inflammation, parenchymal cell injury, and progressive organ fibrosis due to the secretion of matrix metalloproteinases and cytokines, such as IL‐1β, IL‐6, IL‐8, IL‐18, TGF‐β, TNF‐α.^[^ ^5^, ^6^ ^]^ These cytokines activate immune cells to secrete chemokines and proinflammatory cytokines and trigger the activation of fibroblasts to promote the production and deposition of extracellular matrix.^[^ ^2^, ^7^, ^8^ ^]^ Furthermore, SASP causes expansion of senescence to neighboring cells through paracrine effects.^[^ ^9^ ^]^ Reducing the burden of SnCs was shown to effectively extend lifespan and attenuate the progression of aging‐related diseases.^[^ ^10^ ^]^

In addition to the SASP, SnCs are also characterized by persistent DNA damage, cell cycle arrest, oxidative stress, metabolic and epigenetic alterations, and resistance to apoptosis.^[^ ^11^ ^]^ DNA damage is a common cause of senescence.^[^ ^12^ ^]^ Repetitive stressors such as aging, irradiation, and cytotoxic compounds, elicit double‐stranded DNA breaks that activate the DNA damage responsepathway, ultimately causing cell cycle arrest and senescence.^[^ ^13^ ^]^ In SnCs, oxidative stress, which is manifested as high levels of reactive oxygen species (ROS), contributes to DNA damage. SnCs also show metabolic changes, such as increased activity of the senescence‐associated β‐galactosidase (SA‐β‐gal).^[^ ^5^ ^]^ DNA methylation and histone modifications, including methylation and acetylation, are also dysregulated in SnCs.^[^ ^14^ ^]^ For example, histone H3K9 hyperacetylation, mediated by the loss of SIRT6, induces senescence in vascular smooth muscle cell and atherosclerosis during aging.^[^ ^15^ ^]^

The PRDF1 and RIZ1 homology domain (PRDM) family proteins are potent transcriptional regulators.^[^ ^16^ ^]^ These proteins contain a conserved N‐terminal PR domain that enables them to mediate histone methylation, and zinc finger domains that allow them to directly bind to promoters and function as transcription factors (TFs).^[^ ^17^ ^]^ Furthermore, PRDMs act as co‐regulators with other TFs and multiple histone‐modifying proteins, such as histone lysine methyltransferases, histone deacetylases, and histone acetyltransferases.^[^ ^18^ ^]^ Recent studies have suggested that PRDMs are critical to DNA damage repair, fat metabolism, and epigenetic modification.^[^ ^16^, ^19^, ^20^ ^]^ However, the role of PRDMs in cellular senescence has not been elucidated.

In this study, we aimed to determine the role of PRDMs in cellular senescence. We found that PRDM16 decreased significantly in aged organs compared to other PRDMs. Knockout of PRDM16 induced cellular senescence and accelerated organ aging in naturally aged mice. Tubular‐specific *Prdm16* knockout aggravated aging‐related kidney disease. *PRDM16* gene delivery by lentivirus attenuated cellular senescence in vitro and in vivo. Mechanistically, PRDM16 prevented oxidative DNA damage by improving glutathione metabolism. Specifically, PRDM16 upregulated the expression of GSTM1 by binding to its promoter. Collectively, we identified a novel target for anti‐aging interventions.

## 2 Results

### 2.1 PRDM16 is Decreased in Aged Organs and SnCs

**Figure 1:**
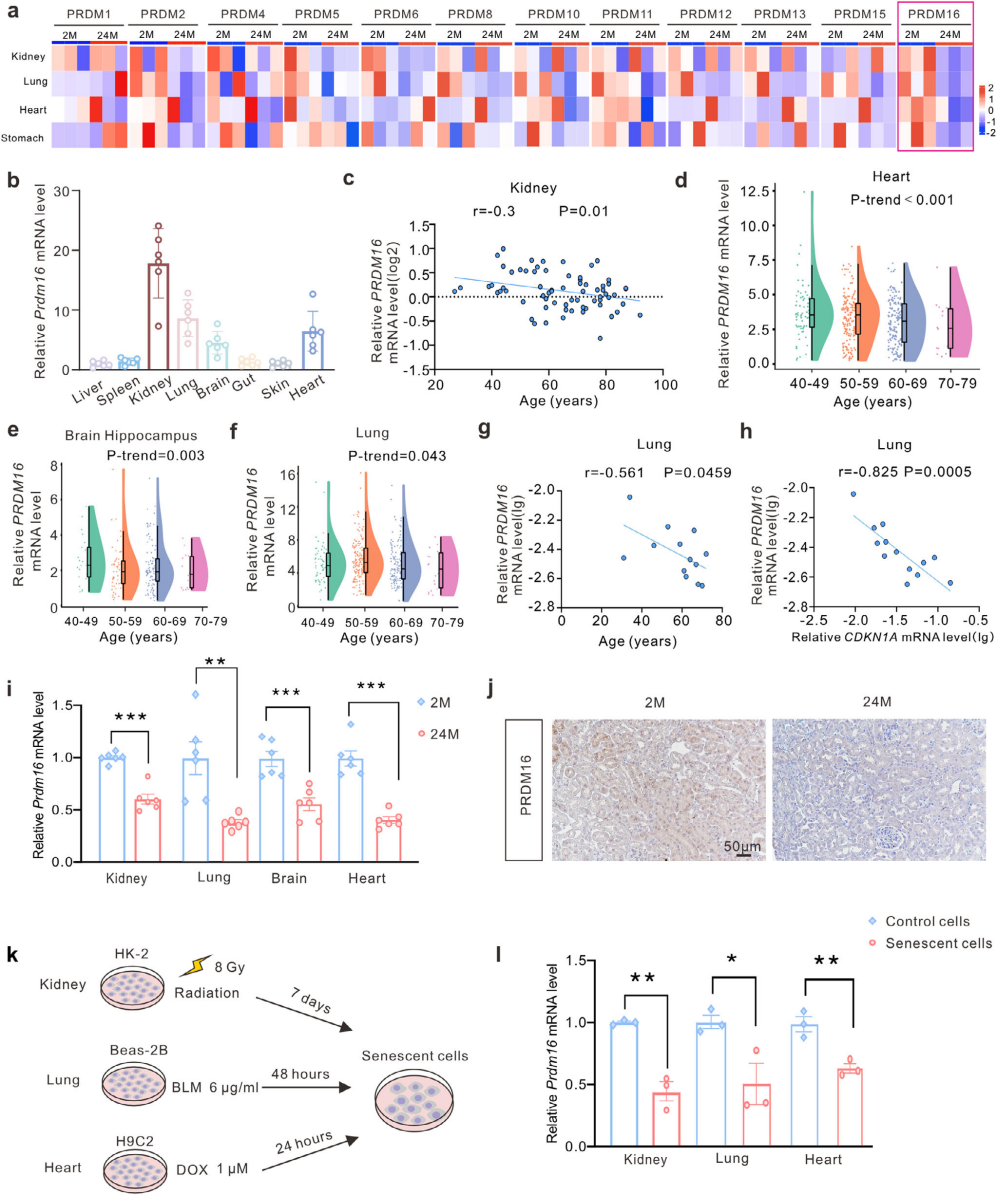
PRDM16 was decreased in aged organs and SnCs. a) qPCR analysis of PRDM family members in the kidneys, lungs, heart and stomach of young (2 months old) and aged (24 months old) mice (n = 3). b) qPCR analysis of *Prdm16* in multiple organs of 2 months mice (n = 6). c) Analysis of one published transcriptomic dataset showed that *PRDM16* decreased with age in human normal renal cortex (n = 71). d–f) Tests for linear trend were conducted between age and *PRDM16* mRNA level in the heart (d) (n = 384), brain hippocampus (e) (n = 187) and lung (f) (n = 345) of humans based on transcriptomes from ADEIP. g) Correlational analysis of *PRDM16* mRNA level (log_10_(2^−(ΔΔCt)^)) and age in human normal lung samples (n = 13). h) Correlational analysis of the mRNA levels (log_10_(2^−(ΔΔCt)^)) of *PRDM16* and *CDKN1A* in human normal lung samples (n = 13). i) qPCR analysis of *Prdm16* in the kidneys, lungs, brain hippocampus and heart of 2 months old (2 M) and 24 months old (24 M) mice (n = 6). j) Representative immunohistochemical (IHC) staining images of PRDM16 in the kidney of 2 months and 24 months mice. Scale bar: 50 µm. k) Diagram detailing X‐ray radiation, BLM and DOX induced cellular senescence. l) qPCR analysis of *Prdm16* in senescent HK‐2, Beas‐2B and H9C2 cells (n = 3). Data are mean ± SEM. *p < 0.05, **p < 0.01 and ***p < 0.001. Spearman's correlations (c, g and h), Test for linear trend (d–f), Two‐tailed Student's unpaired *t*‐test analysis (i and l).

To clarify the expression pattern of PRDM family members during aging, we first performed mRNA analysis in the kidneys, lungs, heart, and stomach of young (2 months old) and aged (24 months old) mice. Our results indicated that only PRDM16 expression exhibited a similar pattern of decrease in multiple aged organs (**Figure** **1**a and Extended Data Table S1, Supporting Information). PRDM16 was expressed in various organs of 2‐month‐old mice, with the highest levels of expression in the kidneys (Figure 1b). Next, we investigated the correlation between *PRDM16* expression levels and age in healthy human kidneys using a human renal cortex transcriptomic dataset from the NephroSeq repository.^[^ ^21^ ^]^ Notably, *PRDM16* levels in the renal cortex were negatively correlated with age (Figure 1c). Then, we assessed the linear trends between age and *PRDM16* mRNA level using transcriptome data from the heart, brain hippocampus, lungs, liver, and stomach of four different age groups from the ADEIP platform.^[^ ^22^ ^]^ Our results showed that in all evaluated organs, except the liver, the levels of *PRDM16* were negatively related to age (Figure 1d–f, Extended Data Figure S1, Supporting Information). Moreover, in normal human lung tissues, *PRDM16* mRNA levels were negatively correlated with both age and the senescence marker *CDKN1A* (Figure 1g,h).

Downregulation of *Prdm16* in the kidneys, lungs, hippocampus, and heart of aged mice was also determined by mRNA analysis (Figure 1i). The level of PRDM16 in aged kidneys was further confirmed by immunohistochemistry (IHC) staining and western blotting (Figure 1j, Extended Data Figure S2, Supporting Information). in vitro, four common methods were utilized to induce cellular senescence, which included X‐ray irradiation and D‐galactose (D‐gal) treatment induced senescence in HK‐2 cells, bleomycin sulfate (BLM) treatment induced senescence in Beas‐2B cells, and doxorubicin hydrochloride (DOX) treatment induced senescence in H9C2 cells (Figure 1k, Extended Data Figure S3a–m, Supporting Information). Our analysis showed that the mRNA and protein levels of PRDM16 were reduced in the senescent HK‐2, Beas‐2B, and H9C2 cells (Figure 1l, Extended Data Figure S3n–p, Supporting Information).

### 2.2 Genetic Deletion of *Prdm16* in Mice Increases Cellular Senescence in Multiple Organs

**Figure 2:**
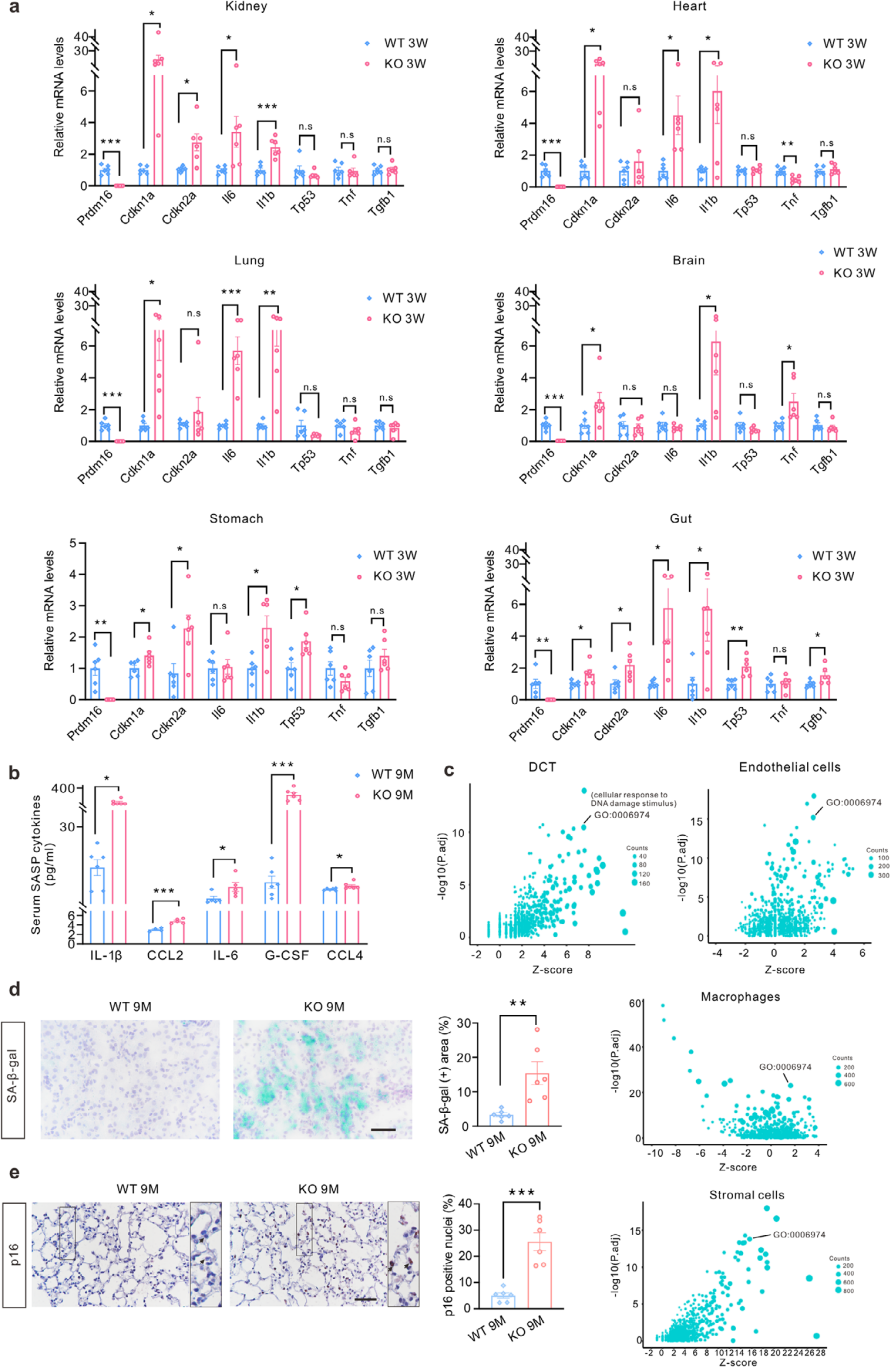
Genetic deletion of *Prdm16* contributed to senescence in multiple organs. a) qPCR analysis of *Prdm16*, senescence and senescence‐associated secretory phenotype (SASP) markers in multiple organs including kidney, heart, lung, brain hippocampus, stomach, and gut of 3 weeks wide type (WT) and global *Prdm16* knockout (KO) mice (n = 6). b) Serum SASP cytokines were measured in 9‐month‐old (9 M) WT and KO mice (n = 6). c) Single‐cell RNA sequencing was conducted with the kidney tissues obtained from 9‐month‐old WT and KO mice (n = 3). A bubble diagram illustrating the enrichment of gene ontology (GO) terms derived from differentially expressed genes in DCT, endothelial cells, macrophages, and stromal cells. d) Representative images of senescence‐associated‐β‐galactosidase (SA‐β‐gal) staining in the kidney. The percentage of SA‐β‐gal positive area was calculated (n = 6). Scale bar: 50 µm. e) Representative IHC staining images of p16 in the lung. The percentage of p16 positive nuclei is calculated (n = 6). Scale bar: 50 µm. Data are mean ± SEM. *p < 0.05, **p < 0.01 and ***p < 0.001. n.s, not significant. Two‐tailed Student's unpaired *t*‐test analysis (a, b, d and e).

To investigate the role of PRDM16 in senescence, we generated *Prdm16* global knockout (KO) mice (Extended Data Figure S4a–c, Supporting Information) and assessed the expression of various senescence markers in these mice. We found that *Prdm16* knockout induced the upregulation of the senescence markers, including *Cdkn1a*, *Cdkn2a*, and *Tp53*, and the SASP related genes, such as *Il6*, *Il1b*, *Tnf*, and *Tgfb1* in multiple organs of 3‐week‐old mice (**Figure** **2**a). The results of SASP cytokines panel showed that serum levels of IL‐1β, CCL2, IL6, G‐CSF, and CCL4 in 9‐month‐old *Prdm16* KO mice were significantly higher than those in wild type (WT) mice (Figure 2b). To better understand the expression profile regulated by PRDM16, we conducted single‐cell RNA sequencing (scRNA‐seq) of the renal cortex of 9‐month‐old WT and *Prdm16* KO mice (Extended Data Figures S5 and S6a, Supporting Information). The identified differentially expressed genes were found to be significantly enriched in DNA damage (GO:0 006974), a characteristic alteration associated with senescence, across various cell types, including the distal convoluted tubule (DCT) cells, endothelial cells, macrophages, and stromal cells (Figure 2c). Moreover, differentially expressed genes were enriched in multiple aging and senescence related Gene Ontology (GO) terms across several cell types (Extended Data Figure S6b, Supporting Information). *Prdm16* deficiency affected the expression of various senescence and DNA damage associated genes, particularly in the proximal convoluted tubules (PCT) and DCT (Extended Data Figure S6c,d, Supporting Information). The expression levels of SA‐β‐gal and senescence markers p21 and Bcl‐2 were also increased in the kidneys of *Prdm16* KO mice (Figure 2d and Extended Data Figure S4d,e, Supporting Information).

Moreover, the proportion of p16‐positive SnCs in the lungs of *Prdm16* KO mice also exhibited an increase (Figure 2e). And the expression of p21 was also elevated in the lungs of *Prdm16* KO mice (Extended Data Figure S4f,g, Supporting Information). These data indicate PRDM16 deficiency induces senescence in multiple organs.

### 2.3 Global Knockout of *Prdm16* Mice Manifests as Progressive Organ Injury at 9 Months of Age

**Figure 3:**
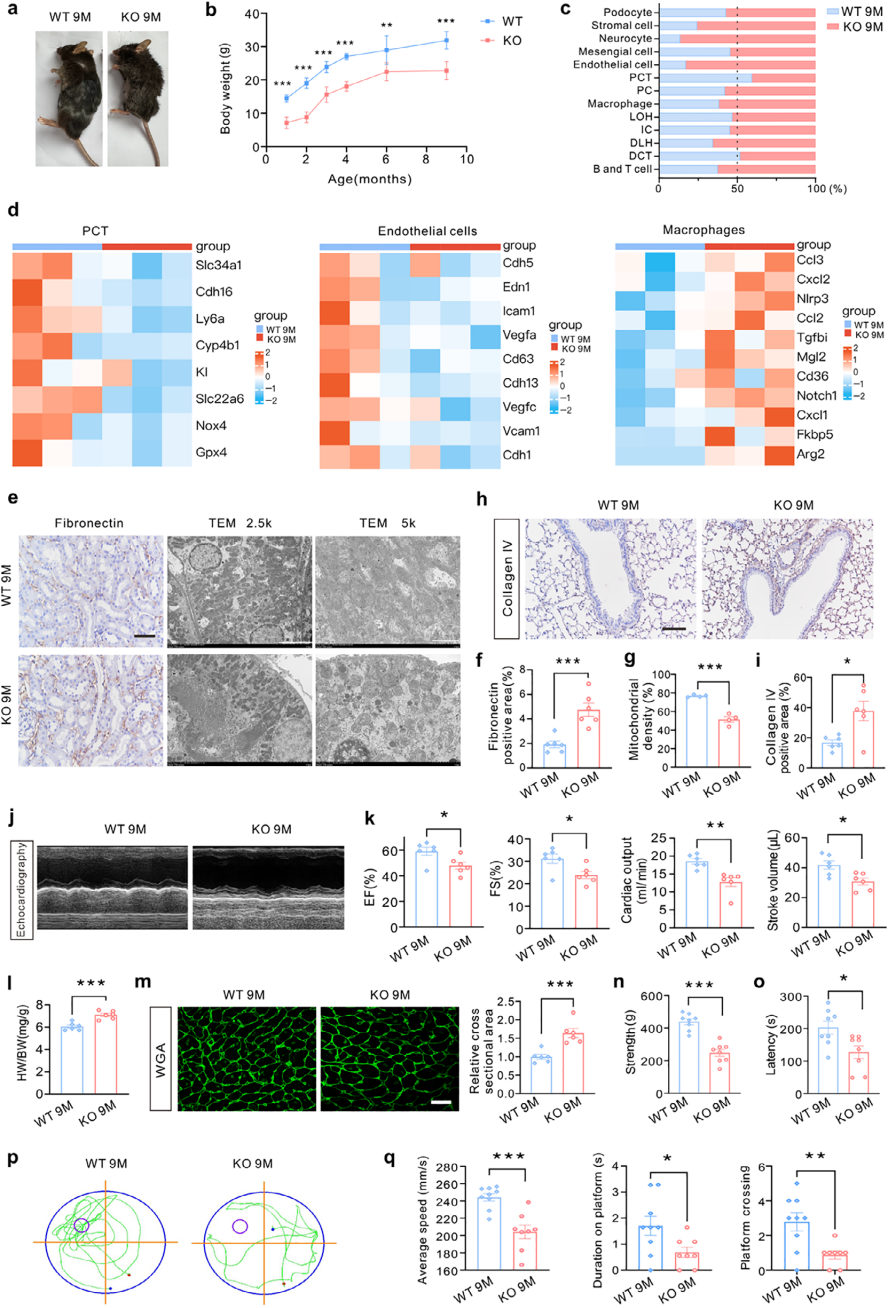
Global knockout of *Prdm16* mice manifested as progressive organ injury at 9 months old. a) Gross appearance of wide type (WT) and *Prdm16* knockout (KO) mice (9 months old). b) Body weight curve from the first month to the ninth month (n = 6). c) Single‐cell RNA sequencing (scRNA‐seq) analysis was performed using renal cortex of WT and *Prdm16* KO mice. Bar‐plot was generated to show the proportion of annotated cell types (n = 3). d) Heatmaps of representative differentially expressed genes in proximal convoluted tubule (PCT), endothelial cells and macrophages based on scRNA‐seq analysis (n = 3). e) Representative IHC staining images of Fibronectin in the kidney and transmission electron microscopy (TEM) images in the tubular epithelial cells of mice. Scale bar: 50 µm (Fibronectin), 5 µm (TEM 2.5k) and 2 µm (TEM 5k). f) The percentage of Fibronectin positive area was calculated (n = 6). g) Mitochondrial density in tubular epithelial cells of mice was calculated (n = 4). h,i) Representative IHC staining images of Collagen IV in the lung (h). Scale bar: 100 µm. The percentage of Collagen IV positive area was calculated (i) (n = 6). j) Representative images of M‐mode echocardiograms. k) Quantitative analysis of ejection fraction (EF), fractional shortening (FS), cardiac output, and stroke volume (n = 6). l) Heart weight to body weight (HW/BW) was calculated (n = 6). m) Representative IF images of wheat germ agglutinin (WGA) staining in the heart. The relative cross‐sectional area of cardiomyocytes was calculated (n = 6). Scale bar: 25 µm. n) Strength analysis of the forelimb grip strength test (n = 8). o) Latency analysis of the rotarod test (n = 8). p) Swimming track in the Morris water maze (MWM) test. q) Analysis of average speed, duration on platform and the number of times crossing platform in mice (n = 9 for WT group, n = 8 for KO group). Data are mean ± SEM. *p < 0.05, **p < 0.01 and ***p < 0.001. Two‐tailed Student's unpaired it‐test analysis (b, f, g, i, k–o and q).

Compared to the WT mice, *Prdm16* KO mice at 9 months of age displayed a smaller appearance and lower body weight (**Figure** **3**a,b). Under identical housing conditions, elder *Prdm16* KO mice (9 months old) showed an increased fecal load accumulation rate comparable to that of aged WT mice (24 months old) (Extended Data Figure S7a, Supporting Information). Multiple organ injuries were also observed. In the kidneys, the proportions of diverse cell types were quite different after *Prdm16* KO as revealed by scRNA‐seq (Figure 3c). Furthermore, the marker genes of healthy PCT and endothelial cells were downregulated in the KO mice, indicating cellular injury within these populations. However, macrophages were activated in the kidneys of *Prdm16* KO mice (Figure 3d). IHC for fibronectin revealed a modest increase in the kidneys of *Prdm16* KO mice, suggesting increased susceptibility to fibrosis. Additionally, transmission electron microscopy (TEM) showed structural damage of mitochondria and the decrease of mitochondrial density and length in the tubular epithelial cells of *Prdm16* KO mice (Figure 3e–g, Extended Data Figure S7b,c, Supporting Information). IHC of collagen IV showed an increase in lungs of *Prdm16* KO mice (Figure 3h,i, Supporting Information).

To determine the role of PRDM16 in cardiac injury, cardiac function was assessed using echocardiography. *Prdm16* KO mice exhibited a noticeable decline in ejection fraction (EF), fractional shortening (FS), cardiac output, and stroke volume compared to WT mice (Figure 3j,k, Supporting Information). However, the heart weight‐to‐body weight ratio (HW/BW) and the cardiomyocyte cross‐sectional area of *Prdm16* KO mice were higher than those of WT mice (Figure 3l,m, Supporting Information). The forelimb grip strength test indicated poorer muscle function in *Prdm16* KO mice than in WT mice (Figure 3n). The rotarod test results demonstrated impaired locomotor ability in *Prdm16* KO mice compared to the ability of WT mice (Figure 3o). Besides, the Morris water maze (MWM) test was employed to assess spatial learning and memory capabilities. *Prdm16* KO mice exhibited slower swimming speed, reduced duration on the platform, and decreased frequency of crossing the platform compared to WT mice, indicating poor spatial learning and memory abilities (Figure 3p,q). Hematoxylin‐eosin (H&E) staining of the liver and skin revealed lipid droplet deposition in the liver and a reduced number of hair follicles in the skin of *Prdm16* KO mice (Extended Data Figure S7d,e, Supporting Information).

### 2.4 Tubule‐Specific *Prdm16* Deletion Aggravates Aging‐Related Kidney Diseases

We further investigated the role of PRDM16 in aging‐related kidney diseases, considering that the kidneys exhibited the highest expression level of PRDM16. The subcellular localization of PRDM16 in the kidneys was predominantly observed in tubules (Figure 1j). Consequently, tubule‐specific *Prdm16* knockout (*Ksp‐Cre/Prdm16^fl/fl^*) mice were generated using the Cre‐LoxP recombination system, as confirmed by mRNA analysis of the kidneys and other organs (Extended Data Figure S8a–c, Supporting Information). The liver function, kidney function, and blood glucose levels of *Ksp‐Cre/Prdm16^fl/fl^* mice were identical to *Prdm16^fl/fl^* mice at 8 weeks of age (Extended Data Figure S8d–h, Supporting Information). In addition, H&E staining revealed that the structures of the heart, kidneys, perirenal fat, lungs, spleen, and liver in *Ksp‐Cre/Prdm16^fl/fl^* mice were comparable to those in *Prdm16^fl/fl^* mice (Extended Data Figure S8i–k, Supporting Information).

**Figure 4:**
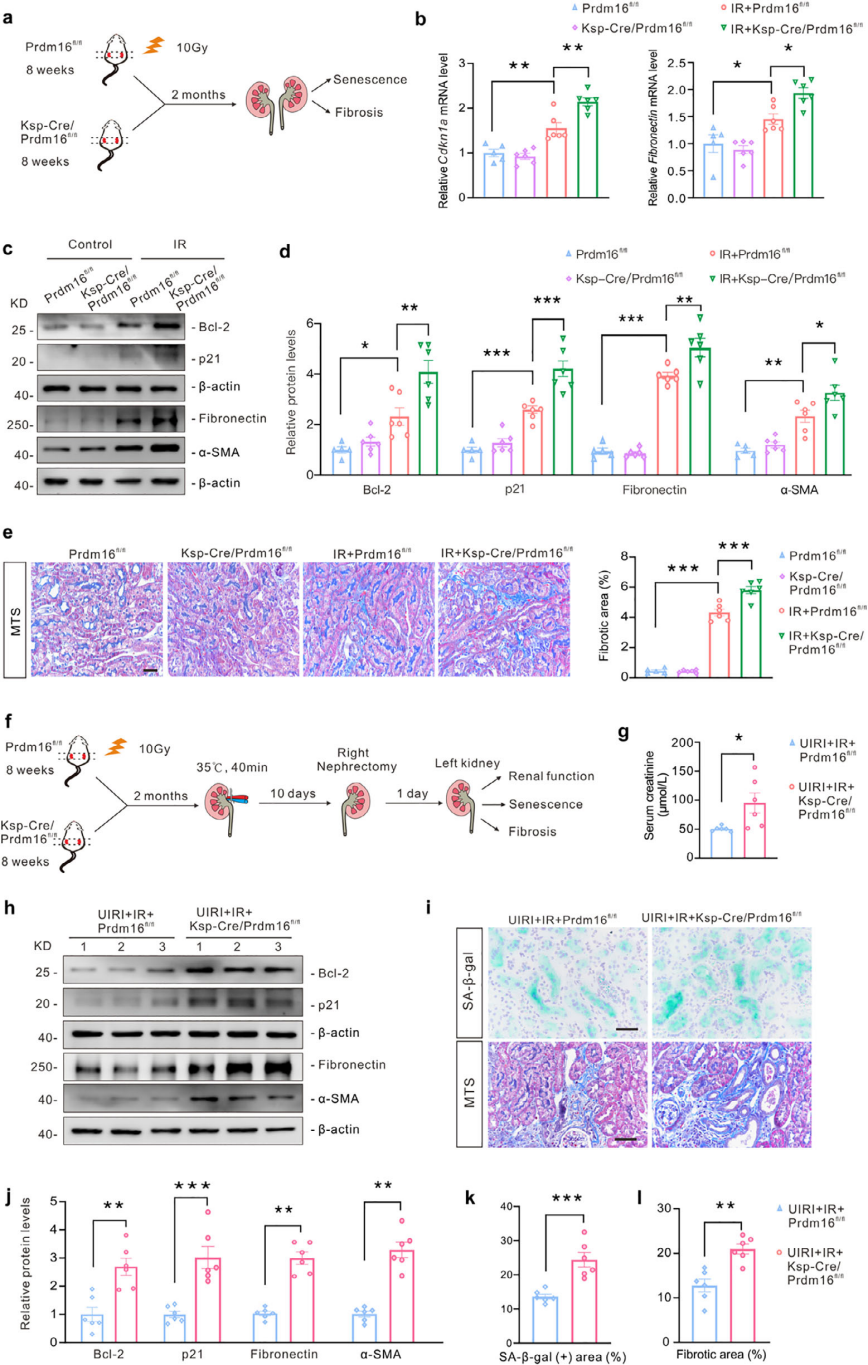
Tubule‐specific *Prdm16* deletion aggravated aging‐related kidney diseases. a) Diagram detailing irradiation (IR) in *Prdm16^fl/fl^* mice and *Ksp‐Cre*/*Prdm16^fl/fl^* mice. b) qPCR analysis of *Cdkn1a* and *Fibronectin* in the kidney cortex (n = 5 for *Prdm16^fl/fl^* group, n = 6 for the other 3 groups). c,d) Representative western blot images (c) and quantification (d) of senescence and fibrotic markers in the kidney cortex (n = 5 for *Prdm16^fl/fl^* group, n = 6 for the other 3 groups). e) Representative images of Masson's trichrome staining (MTS) in the kidney. The percentage of fibrotic area was calculated (n = 5 for *Prdm16^fl/fl^* group, n = 6 for the other 3 groups). Scale bar: 50 µm. f) Diagram detailing unilateral ischemia reperfusion injury (UIRI) surgery in irradiated mice. g) Serum creatinine in mice (n = 6). h,j) Representative western blot images (h) and quantification (j) of senescence and fibrotic markers in the kidney cortex (n = 6). Numbers (1–3) represent different animals in a given group. i) Representative images of SA‐β‐gal staining and MTS in the kidney. Scale bar: 50 µm. k,l) The percentages of SA‐β‐gal positive area (k) and fibrotic area (l) were calculated (n = 6). Data are mean ± SEM. *p < 0.05, **p < 0.01 and ***p < 0.001. One‐way ANOVA followed by Tukey's post‐test (b, d and e), two‐tailed Student's unpaired it‐test analysis (g and j–l).

Kidney aging was induced in *Prdm16^fl/fl^* and *Ksp‐Cre/Prdm16^fl/fl^* mice by X‐ray irradiation (**Figure** **4**a). Control mice, which were housed under identical conditions, did not receive kidney irradiation. Irradiated *Ksp‐Cre/Prdm16^fl/fl^* mice exhibited higher expression of the senescence markers p21 and Bcl‐2, as well as the fibrosis markers fibronectin and α‐SMA, in comparison to the control mice (Figure 4b–d). Additionally, Masson's trichrome staining (MTS) indicated increased collagen deposition in the irradiated kidneys of *Ksp‐Cre/Prdm16^fl/fl^* mice compared to *Prdm16^fl/fl^* mice (Figure 4e).

Unilateral ischemia‐reperfusion (UIRI) surgery was performed on the kidneys following irradiation to evaluate the impact of senescence on the regenerative capacity of the kidneys after injury (Figure 4f). *Ksp‐Cre/Prdm16^fl/fl^* mice showed worse kidney function, higher expression of senescence markers p21 and Bcl‐2, as well as fibrosis markers fibronectin and α‐SMA after UIRI, compared to *Prdm16^fl/fl^* mice (Figure 4g,h,j). Moreover, the expression of SA‐β‐gal and collagen deposition were also increased in the kidneys of *Ksp‐Cre/Prdm16^fl/fl^* mice (Figure 4i,k,l). Taken together, these results show that the tubule‐specific *Prdm16* deletion aggravates irradiation‐induced kidney aging and accelerates UIRI‐induced kidney disease under aging conditions.

### 2.5 Lentivirus‐Mediated Delivery of Exogenous *PRDM16* Gene Mitigates Senescence and Ameliorates Renal Aging

**Figure 5:**
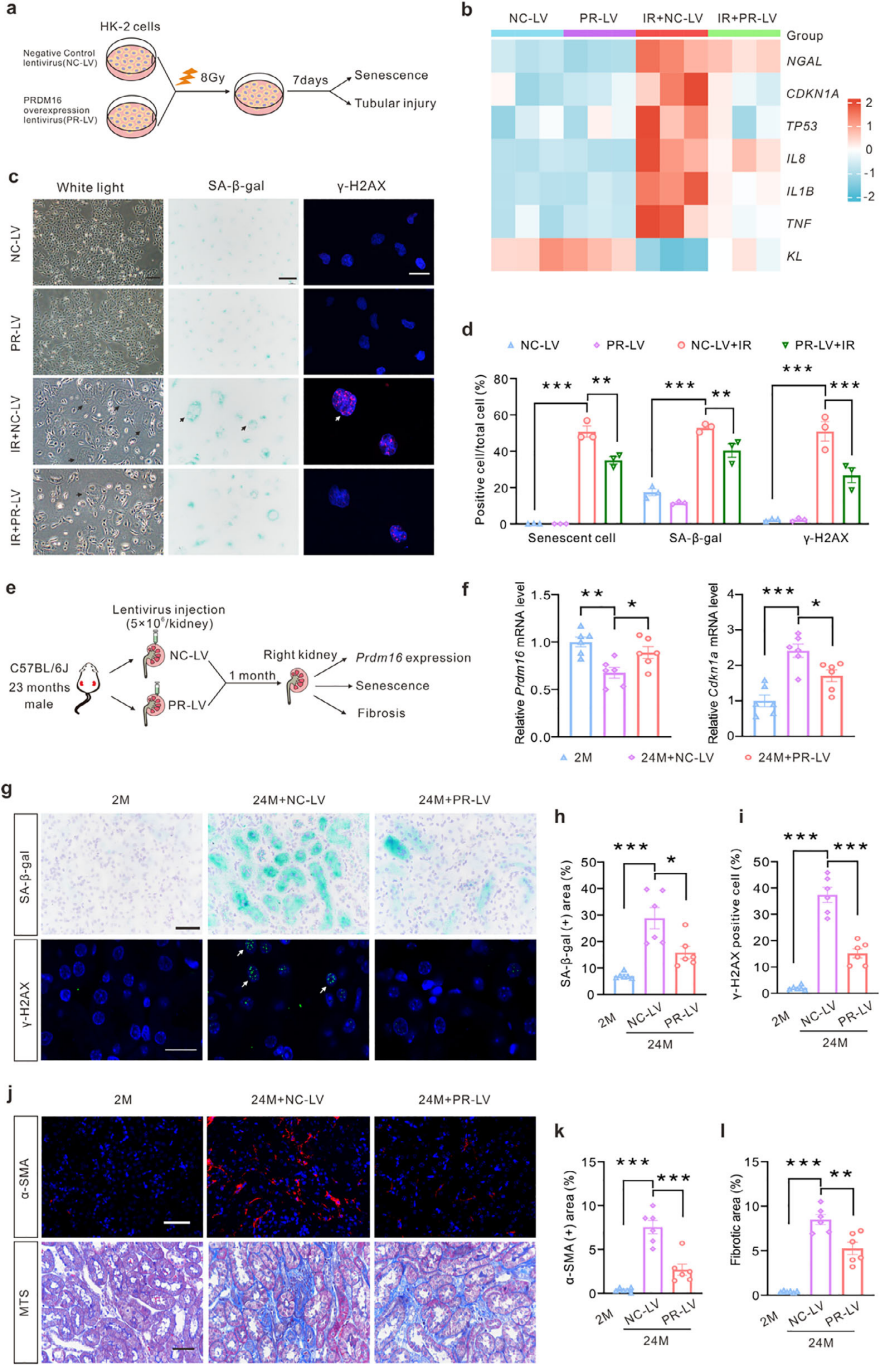
Lentivirus‐mediated delivery of exogenous *PRDM16* gene mitigated cellular senescence and ameliorated renal aging. a) Diagram detailing X‐ray irradiation (IR) induced cellular senescence in HK‐2 cells transfected with negative control lentivirus (NC‐LV) or *PRDM16* overexpression lentivirus (PR‐LV). b) qPCR analysis of tubular injury, senescence and SASP related genes displayed by a heatmap (n = 3). c) Microscopic images depicted morphological changes of HK‐2 cells. Representative images of SA‐β‐gal staining and IF images of γ‐H2AX. Scale bar: 200 µm (left panel), 50 µm (middle panel) and 20 µm (right panel). The arrows pointed to positive cells. d) The percentages of morphologically altered senescent cells, SA‐β‐gal positive cells and γ‐H2AX positive cells in total cells were calculated (n = 3). e) Diagram detailing renal cortex lentivirus injection in old mice. f) qPCR analysis of *Prdm16* and *Cdkn1a* in the kidney cortex (n = 6). g) Representative images of SA‐β‐gal staining and IF images of γ‐H2AX in the kidney. Scale bar: 50 µm (top panel) and 10 µm (bottom panel). h,i) The percentages of SA‐β‐gal (h) and γ‐H2AX (i) positive area were calculated (n = 6). j) Representative IF images of α‐SMA and images of MTS in the kidney. Scale bar: 50 µm. k,l) The percentages of α‐SMA positive area (k) and fibrotic area (l) were calculated (n = 6). Data are mean ± SEM. *p < 0.05, **p < 0.01 and ***p < 0.001. One‐way ANOVA followed by Tukey's post‐test (d, f, h, i, k and l).

To investigate the anti‐senescence effects of PRDM16, we transfected HK‐2 cells with lentivirus overexpressing *PRDM16* and subsequently induced senescence through X‐ray irradiation (**Figure** **5**a and Extended Data Figure S9a, Supporting Information). The mRNA heatmap analysis demonstrated that transfection with *PRDM16* mitigated cell injury and senescence in irradiated HK‐2 cells (Figure 5b). Irradiated HK‐2 cells exhibited typical morphological characteristics of SnCs, including a notable increase in cell body and nucleus volume, as well as a flattened cell shape. PRDM16 significantly improved these morphological changes and reduced the levels of SA‐β‐gal, and DNA damage as revealed by γ‐H2AX staining induced by irradiation (Figure 5c and 5d). Furthermore, transfection with *PRDM16* also suppressed the protein expression of senescence and SASP markers induced by irradiation (Extended Data Figure S9b–e, Supporting Information). In a D‐gal‐induced senescence model of HK‐2 cells, PRDM16 similarly alleviated the upregulation of senescence and SASP markers (Extended Data Figure S9f–l, Supporting Information).

To investigate the impact of PRDM16 on kidney aging in vivo, *PRDM16* overexpression or negative control lentivirus was injected into the renal cortex of aged mice (23 months old) (Figure 5e). IHC staining and mRNA analysis revealed higher levels of PRDM16 in the kidneys of the aged mice injected with the PRDM16‐overexpression lentivirus than in mice injected with the negative control lentivirus (Figure 5f, Extended Data Figure S10a,b, Supporting Information). The expression levels of the senescence markers Bcl‐2 and p21, SA‐β‐gal and γ‐H2AX were elevated in the kidneys of 24‐month‐old mice. However, PRDM16 reduced the expression of these markers (Figure 5f–i, Extended Data Figure S10c, Supporting Information). Moreover, the expression levels of the fibrosis markers fibronectin and α‐SMA, as well as collagen deposition, were increased in the kidneys of aged mice, whereas PRDM16 effectively mitigated these markers of kidney fibrosis (Figure 5j–l, Extended Data Figure S10d, Supporting Information).

### 2.6 PRDM16 Inhibits Oxidative DNA Damage and Improves Glutathione Metabolism

**Figure 6:**
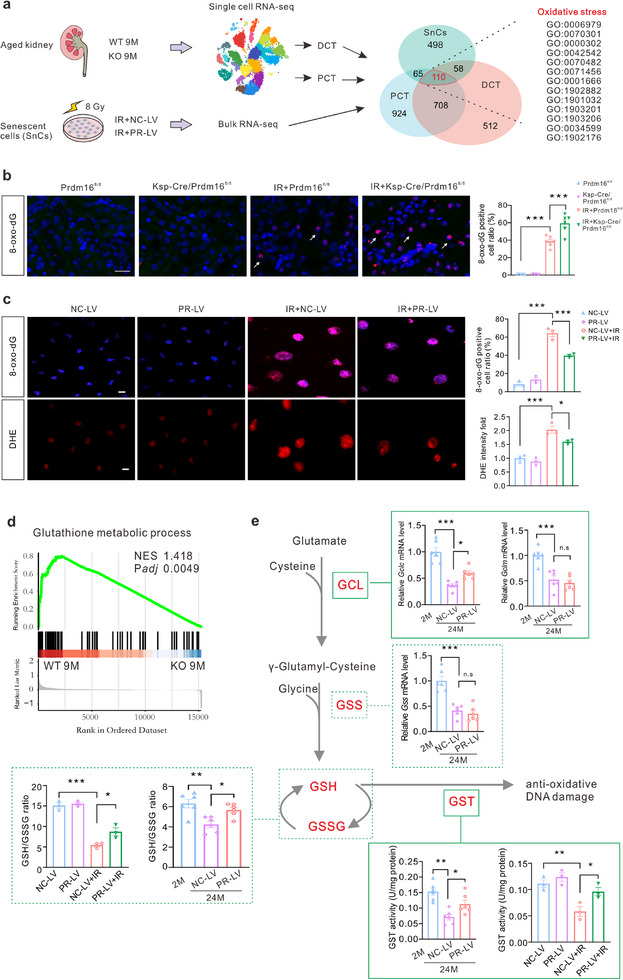
PRDM16 inhibited oxidative DNA damage and improved glutathione metabolism. a) Diagram detailing combined analysis of kidney scRNA‐seq data from 9‐month‐old WT and *Prdm16* KO mice (n = 3) and bulk RNA sequencing (bulk RNA‐seq) data from irradiated PRDM16‐overexpressing and control HK‐2 cells (n = 3). Gene Ontology (GO) enrichment analysis was performed to differentially expressed genes of proximal convoluted tubule (PCT) and distal convoluted tubule (DCT) from scRNA‐seq, as well as differentially expressed genes of HK‐2 cells from RNA‐seq. Common GO terms were identified by intersection. b) Representative IF images of 8‐oxo‐dG in the kidney. The ratio of 8‐oxo‐dG positive cells to total cells was calculated (n = 5 for *Prdm16^fl/fl^* group, n = 6 for the other 3 groups). Scale bar: 20 µm. c) Representative IF images of 8‐oxo‐dG and Dihydroethidium (DHE) in HK‐2 cells. The percentage of 8‐oxo‐dG positive cells in total cells and relative fold change of DHE intensity were calculated. Scale bar: 20 µm. d) Enrichment plot from the GSEA results based on scRNA‐seq data of PCT. e) Diagram detailing the enzymes involved in glutathione metabolism. qPCR analysis of *Gclc*, *Gclm* and *Gss* in the kidney of aging mice treated with negative control lentivirus (NC‐LV) or PRDM16 overexpression lentivirus (PR‐LV). GSH/GSSG ratio and GST activity of irradiated HK‐2 cells and the kidney of aging mice were detected and calculated. (n = 3 for HK‐2 cells and n = 6 for mice). Data are mean ± SEM. *p < 0.05, **p < 0.01 and ***p < 0.001. n.s: not significant. One‐way ANOVA followed by Tukey's post‐test (b, c and e).

To further elucidate the mechanism by which PRDM16 regulates senescence, we conducted a combined analysis of kidney scRNA‐seq data from 9‐month‐old WT and *Prdm16* KO mice and bulk RNA sequencing (bulk RNA‐seq) data from senescent PRDM16‐overexpressing and control HK‐2 cells induced by irradiation. GO enrichment analysis was performed on differentially expressed genes in PCT and DCT from the scRNA‐seq data, and on the differentially expressed genes identified in the RNA‐seq data. Shared GO terms were identified by intersecting GO terms from different sources, revealing a significant enrichment of terms related to oxidative stress (**Figure** **6**a). This finding suggests that PRDM16 may regulate senescence by modulating oxidative stress. DNA damage, primarily resulting from oxidative stress, is a major driver of cellular senescence.^[^ ^23^ ^]^ Therefore, we measured the levels of 8‐oxo‐dG, a well‐established marker of oxidative DNA damage.^[^ ^24^, ^25^ ^]^ In *Prdm16^fl/fl^* mice exposed to irradiation, the expression of 8‐oxo‐dG in the kidneys was upregulated. Tubule‐specific *Prdm16* deletion exacerbated the elevation of 8‐oxo‐dG (Figure 6b). In irradiated HK‐2 cells, the levels of 8‐oxo‐dG and ROS were both elevated. However, PRDM16 transfection alleviated oxidative stress induced by irradiation (Figure 6c). According to the enrichment plot of the gene set enrichment analysis (GSEA) results, a number of genes enriched in the pathway “Glutathione metabolic process” were decreased in the PCT of *Prdm16* KO mice (Figure 6d). The GO enrichment analysis and differentially expressed gene analysis indicated that PRDM16 affected glutathione metabolism across different cell types (Extended Data Figure S11, Supporting Information). And *Prdm16* deficiency downregulated the expression of various glutathione metabolism associated genes in the PCT and DCT (Extended Data Figure S12, Supporting Information). Given the importance of glutathione metabolism in antioxidant defense, these results suggest that PRDM16 may mitigate senescence by enhancing resistance to oxidative stress through its role in regulating glutathione metabolism.^[^ ^26^ ^]^ During glutathione metabolism, glutamate cysteine ligase (GCL) and glutathione synthetase (GSS) catalyze the sequential combination of intracellular glutamate with cysteine and glycine to form glutathione (GSH).^[^ ^27^ ^]^ GSH undergoes reactions for ROS detoxification catalyzed by glutathione S‐transferase (GST) or other molecules, thereby exerting antioxidant effects against DNA damage induced by oxidative stress.^[^ ^12^, ^28^, ^29^ ^]^ The mRNA levels of *Gclc*, *Gclm*, and *Gss* were downregulated in the kidneys of aged mice, whereas PRDM16 specifically upregulated the mRNA level of *Gclc*. Transfection with PRDM16 effectively increased the GSH/GSSG ratio in irradiated HK‐2 cells and the kidneys of aged mice. Furthermore, PRDM16 elevated the activity of GST enzyme (Figure 6e). These data suggest that PRDM16 inhibits oxidative DNA damage and improves glutathione metabolism in SnCs.

### 2.7 PRDM16 Functions as a Transcription Activator of GSTM1

**Figure 7:**
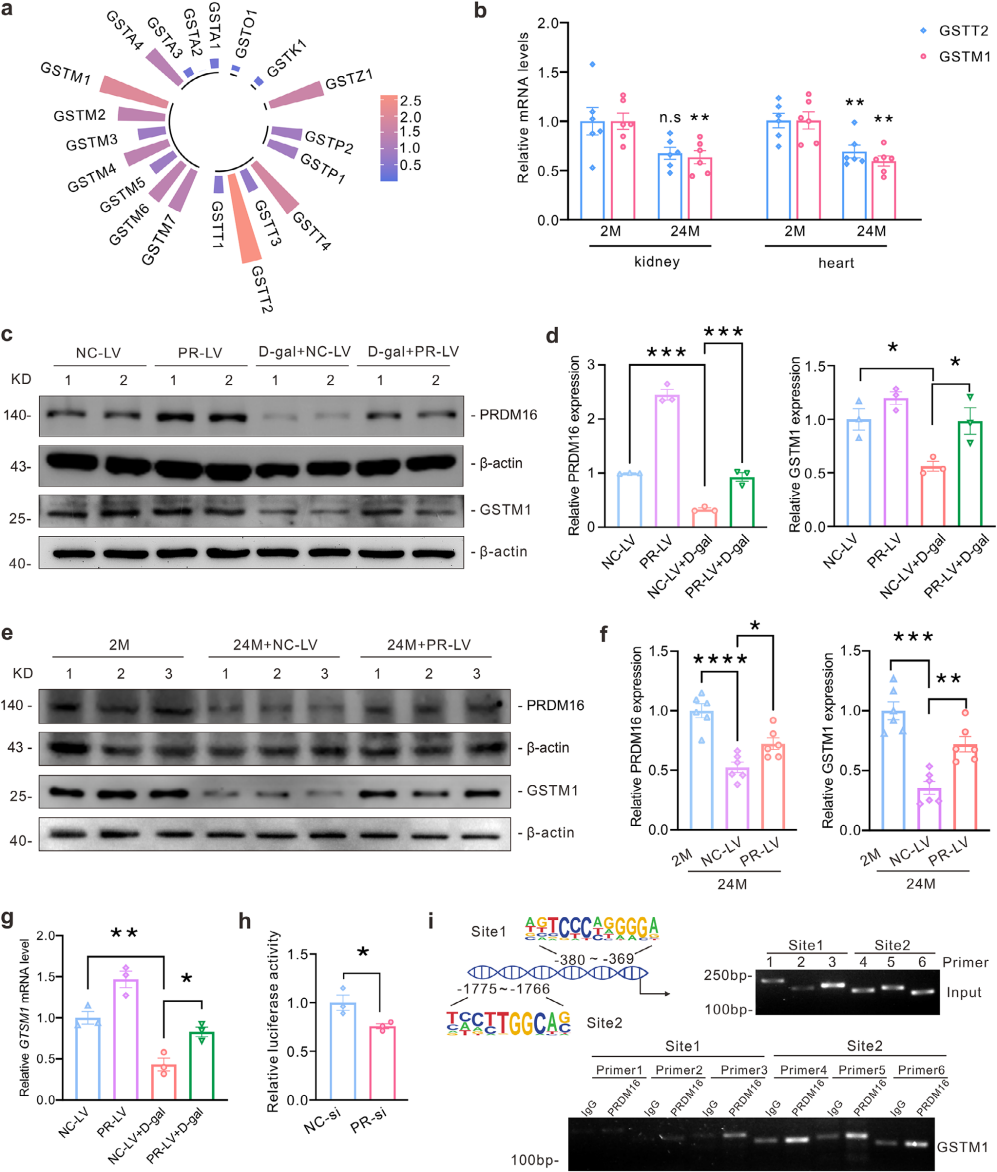
PRDM16 acted as a transcription activator of GSTM1. a) Radiation plot depicting the predicted scores of PRDM16 binding to the promoter region of GST family members based on the chromatin immunoprecipitation sequencing (ChIP‐Seq) data of PRDM16 deposited in the Cistrome DB database. b) qPCR analysis of *Gstt2* and *Gstm1* in the kidney and heart of 2‐month‐old and 24‐month‐old mice (n = 6). c,d) A representative western blot (c) and quantification (d) of PRDM16 and GSTM1 in HK‐2 cells (n = 3). Numbers (1‐2) represent different wells of cells. e,f) A representative western blot (e) and quantification (f) of PRDM16 and GSTM1 in renal cortex (n = 6). Numbers (1–3) represent different animals in the given group. g) qPCR analysis of GSTM1 in HK‐2 cells with different treatments (n = 3). h) HEK‐239T cells were co‐transfected with firefly luciferase reporter plasmid of GSTM1 and Renilla reniformis luciferase reporter plasmid, either negative control siRNA or PRDM16 siRNA. Relative luciferase activity of different groups was measured. i) Two reported PRDM16 binding sites were found in the promoter region of GSTM1. Three primers were designed for each binding site. Representative images of ChIP‐PCR conducted in HK‐2 cells using anti‐PRDM16 and anti‐IgG antibody. Data are mean ± SEM. *p < 0.05, **p < 0.01, ***p < 0.001 and ****p < 0.0001. n.s: not significant. Two‐tailed Student's unpaired *t*‐test analysis (b and h), one‐way ANOVA followed by Tukey's post‐test (d, f and g).

Given the significant enhancement of GST activity by PRDM16 and the pivotal role of GST in executing antioxidative stress within glutathione metabolism, we focused on members of the GST family.^[^ ^29^, ^30^ ^]^ We analyzed chromatin immunoprecipitation sequencing (ChIP‐Seq) data obtained from brown adipose tissue immunoprecipitated with an anti‐PRDM16 antibody, as reported in a published study available in the Cistrome DB database.^[^ ^31^ ^]^ Our analysis showed that PRDM16 specifically bound to the promoter regions of some GST family members, with the promoters of GSTT2 and GSTM1 exhibiting the highest binding scores (**Figure** **7**a). The mRNA analysis revealed a greater decrease in GSTM1 than in GSTT2 levels in both the kidneys and heart of aged mice (Figure 7b). Therefore, further studies were focused on GSTM1. In irradiated or D‐gal‐induced SnCs and the kidneys of aged mice, the expression levels of GSTM1 were decreased (Extended Data Figure S13a–f, Supporting Information). However, PRDM16 upregulated the mRNA and protein levels of GSTM1 (Figure 7c–g). Dual‐luciferase reporter assay showed that PRDM16 silencing reduced the expression from the promoter of GSTM1 (Figure 7h). And PRDM16 silencing reduced the protein expression level of GSTM1 (Extended Data Figure S13g,h, Supporting Information). We scanned the DNA sequence of the GSTM1 promoter for reported PRDM16 binding sites and identified two putative binding sites for PRDM16 within the DNA sequence of GSTM1 promoter.^[^ ^31^ ^]^ A ChIP assay was conducted using PCR primers targeting these putative binding sites, which showed PRDM16 specifically bound to these regions in the GSTM1 promoter (Figure 7i). And consistent results were observed in the kidney of wild type mice but not in global *Prdm16* knockout mice (Extended Data Figure S13i, Supporting Information). These results indicate that PRDM16 functions as a transcriptional activator of GSTM1.

### 2.8 Restoration of GSTM1 Alleviates Cellular Senescence and Renal Aging Induced by PRDM16 Deficiency in vitro and in vivo

**Figure 8:**
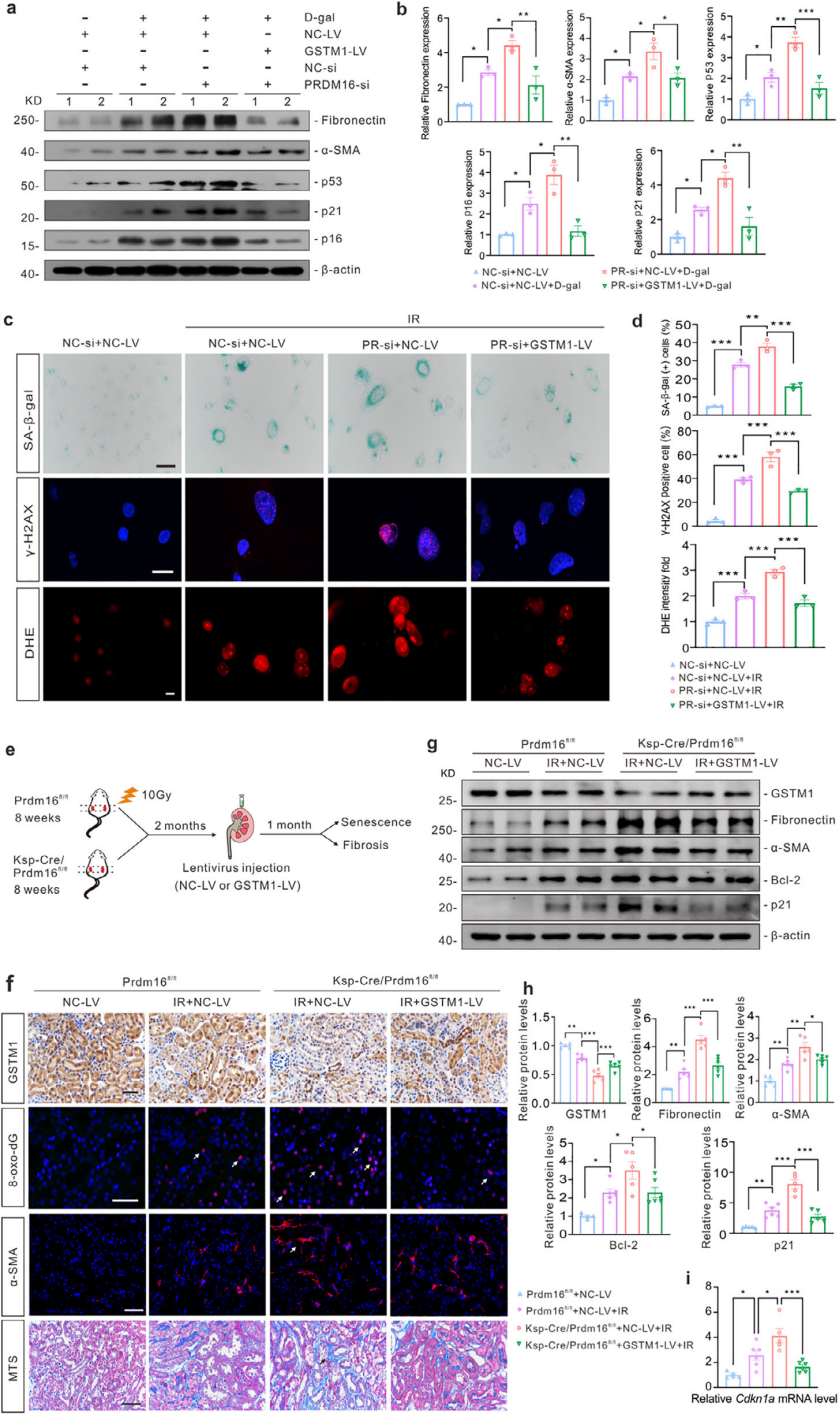
Restoration of GSTM1 alleviated cellular senescence and renal aging induced by PRDM16 deficiency in vitro and in vivo. a,b) A representative western blot (a) and quantification (b) of senescence and fibrotic markers in HK‐2 cells with different treatments (n = 3). NC‐LV, negative control lentivirus. GSTM1‐LV, *GSTM1* overexpression lentivirus. NC‐si, negative control siRNA. PR‐si, PRDM16 siRNA. Numbers (1‐2) represent different wells of cells in a given group. c) Representative images of SA‐β‐gal staining, IF images of γ‐H2AX and DHE staining. Scale bar: 50 µm (top panels) and 20 µm (middle and bottom panel). d) The percentages of SA‐β‐gal positive cells and γ‐H2AX positive cells in total cells were calculated (n = 3). The relative fold change of DHE intensity was quantified (n = 3). e) Diagram detailing kidney lentivirus injection in irradiated mice. f) Representative IHC staining images of GSTM1, IF images of 8‐oxo‐dG and α‐SMA and images of MTS in the kidney. Scale bar: 20 µm (8‐oxo‐dG) and 50 µm (the others). g,h) A representative western blot (g) and quantification (h) of GSTM1, senescence and fibrosis markers in the kidney cortex. (i) qPCR analysis of *Cdkn1a* in the kidney cortex (n = 5 for *Prdm16^fl/fl^* + NC‐LV group and *Ksp‐Cre/Prdm16^fl/fl^* + NC‐LV + IR group, n = 6 for *Prdm16^fl/fl^* + NC‐LV + IR group and *Ksp‐Cre/Prdm16^fl/fl^* + GSTM1‐LV + IR group). Data are mean ± SEM. *p < 0.05, **p < 0.01 and ***p < 0.001. One‐way ANOVA followed by Tukey's post‐test (b, d, h and i).

Although PRDM16 transcriptionally regulates GSTM1, it is unclear whether GSTM1 mediates the effects of PRDM16 on senescence. In D‐gal‐induced SnCs, silencing of PRDM16 increased the expression of senescence and fibrotic markers, whereas transfection with *GSTM1* overexpression lentivirus mitigated the damage caused by PRDM16 silencing (**Figure** **8**a,b). Silencing of PRDM16 also further elevated the level of SA‐β‐gal, γ‐H2AX, and ROS induced by irradiation in vitro, whereas GSTM1 effectively rescued the damage caused by PRDM16 silencing (Figure 8c,d). For the in vivo study, the kidneys of *Prdm16^fl/fl^* and *Ksp‐Cre/Prdm16^fl/fl^* mice were exposed to irradiation, followed by the injection of *GSTM1* overexpression lentivirus or control lentivirus into the renal cortex of these mice (Figure 8e). Western blotting and IHC staining of GSTM1 showed that the expression of GSTM1 was increased after *GSTM1* overexpression lentivirus injection (Figure 8f–h). Aging of the kidneys was also observed. Tubule‐specific *Prdm16* deletion aggravated the expression of senescence markers and 8‐oxo‐dG, as well as kidney fibrosis following irradiation. Conversely, GSTM1 alleviated the progression of senescence and fibrosis caused by PRDM16 deletion (Figure 8f–i).

## 3 Discussion

Our findings demonstrated that PRDM16 expression was decreased in aged organs of humans and mice, as well as in SnCs in vitro. *Prdm16* knockout accelerated aging in multiple organs, including the kidneys, lungs, heart, and brain, and was associated with aging‐related kidney disease. Conversely, delivery of the *PRDM16* gene attenuated cellular senescence and renal aging. The underlying mechanism by which PRDM16 inhibited senescence involved the suppression of oxidative DNA damage through the enhancement of glutathione metabolism. Furthermore, the exogenous expression of GSTM1, a downstream target of PRDM16, mitigated cellular senescence and renal aging. In this study, we elucidated the anti‐senescence role of PRDM16 and its underlying mechanism, providing a potential target for mitigating aging and aging‐related diseases.

PRDM16 was previously shown to be an important regulator of adipocyte differentiation.^[^ ^32^, ^33^ ^]^ However, the role of PRDM16 in senescence remains unclear. The expression of PRDM16 was relatively abundant in solid organs, including the kidneys, lungs, heart, and brain, compared to that in the liver, spleen, skin, and gut. Analysis of human transcriptomic data from Nephroseq database revealed an age‐related decrease in *PRDM16* RNA level in the renal cortex. This finding, however, was not replicated in GTEx dataset (data not shown). Considering the transcriptomic data we analyzed from Nephroseq database was based on a human cohort specifically focused on kidney aging. And it contains specific age for each participant and has a more appropriate male‐to‐female ratio. We thought the transcriptomic data from Nephroseq was a more reliable source for analyzing the expression of PRDM16 in kidney aging in our study. The correlation between PRDM16 expression and age in heart, lung and brain tissues was based on the GTEx dataset. Due to limited samples in the 20–29 and 30–39 age groups, our analysis specifically focused on the correlation between PRDM16 expression and age among individuals aged 40 and above. A significant negative correlation between PRDM16 levels and age was observed in the heart, lungs, brain hippocampus, and stomach; however, no such correlation was observed in the liver.

Subsequently, global *Prdm16* KO mice were generated and confirmed to exhibit cellular senescence in multiple organs, including those with relatively low PRDM16 abundance. Notably, this phenomenon was observed as early as 3 weeks before weaning. However, organ injury was not observed on cardiac ultrasonography, with H&E staining, or in blood biochemistry. These transgenic mice were fed until they reached 9 months of age. At this time point, kidney interstitial fibrosis and inflammation, renal tubular injury, lung fibrosis, decreased cardiac function, myocardial hypertrophy, decreased learning and memorizing abilities, and decreased exercise performance were observed. Interestingly, organs with relatively low PRDM16 expression also exhibited injuries such as lipid deposition in the liver and reduced skin hair follicles in the skin. There are two possible explanations for these observations. First, despite its relatively low expression in some organs, it is important to maintain the biological activity of PRDM16. Second, abundantly expressed PRDM16 in certain tissues is shared with other low‐expressing tissues. The association between PRDM16 and aging‐related fibrosis in the heart and adipose tissues has been previously documented.^[^ ^34^, ^35^ ^]^ The age at which cardiac dysfunction observed in our study was consistent with findings reported in the literature.^[^ ^34^ ^]^ However, glucose tolerance was comparable between WT and *Prdm16* KO mice at 9 months of age.

To confirm the protective role of PRDM16 in senescence, the *PRDM16* gene was delivered to both model senescent cells and mouse kidneys using lentivirus. PRDM16 effectively inhibited cellular senescence in vitro, and ameliorated kidney aging in aged mice. As a prolonged lifespan is compelling evidence supporting the efficacy of anti‐aging strategies,^[^ ^36^, ^37^, ^38^ ^]^ we tried to generate global *Prdm16* knock‐in mice to investigate the impact of PRDM16 on lifespan. However, it is difficult to obtain offspring from these transgenic mice. During mouse embryonic development, the expression of PRDM16 was initially detected in a limited number of tissues on embryonic day 9.5 (E9.5). By E14.5, PRDM16 expression was widespread across various developing tissues, including the brain, lungs, kidneys, and gastrointestinal tract.^[^ ^39^ ^]^ The reason for our failure to generate *Prdm16* knock‐in mice may be that increased PRDM16 expression in the early stages may disturb embryonic development. We plan to attempt to overexpress PRDM16 in mice using the Cre‐LoxP system.

We investigated the mechanism underlying the inhibitory effect of PRDM16 on senescence and found that it involved suppression of oxidative DNA damage. Our study showed, for the first time, the regulatory role of PRDM16 on DNA damage. It was previously reported that PRDM9 is indispensable for the formation of DNA double‐strand breaks.^[^ ^40^ ^]^ During aging, the guanine in the double‐stranded DNA is susceptible to attack by the increased ROS, resulting in its oxidation to 8‐oxo‐7,8‐dihydroguanine (8‐oxo‐dG).^[^ ^41^ ^]^ PRDM16 effectively inhibits the levels of 8‐oxo‐dG and ROS in SnCs. In line with this, PRDM16 deficiency in stem cells leads to elevated ROS levels and subsequent cell death.^[^ ^42^ ^]^ Further investigation revealed that PRDM16 enhances GSH metabolism, a crucial antioxidative stress system.^[^ ^43^ ^]^ Consistently, the PRDM16 mutation diminished GSH levels in cardiac tissues.^[^ ^44^ ^]^ The role of GSH metabolism in the aging process has been discussed. Aged skeletal muscle is characterized by a decreased proportion of GSH‐abundant muscle stem cells. Compared to muscle stem cells with high levels of GSH, those with low levels of GSH exhibit significantly impaired proliferation ability and heightened susceptibility to apoptosis.^[^ ^45^ ^]^ Notably, PRDM16 exclusively upregulated the expression of *Gclc*, a rate‐limiting enzyme, in the biosynthesis pathway of GSH.^[^ ^27^ ^]^ GST activity significantly increased after *PRDM16* gene delivery using a lentivirus. Small extracellular vesicles with intrinsic GST activity from primary fibroblasts of young human donors ameliorate senescence‐related tissue injury.^[^ ^46^ ^]^

We focused our investigation on the GST family protein GSTM1. The expression of GSTM1 was downregulated in aged kidneys and SnCs, whereas PRDM16 enhanced the transcriptional activity of GSTM1 by binding to its promoter region. Additionally, decreased levels of GSTM3, GSTT2, and GSTK1 have been reported in the aged liver tissues.^[^ ^47^ ^]^ The association between GST family members and senescence has been discussed previously.^[^ ^46^, ^48^ ^]^ For example, transfection with recombinant GSTM2 restores the antioxidant and anti‐senescence capacities of small extracellular vesicles derived from old fibroblasts.^[^ ^46^, ^49^ ^]^ Finally, we demonstrated that *GSTM1* gene delivery rescued cellular senescence and kidney aging induced by PRDM16 deficiency. Further robust evidence is required to determine whether GSTM1 represents a viable target for interventions aimed at mitigating the aging process. Based on our findings and emerging literature, we propose the following translational approaches for future investigation. Gene therapy has advanced rapidly over the past two decades, achieving successes in both preclinical studies and clinical applications. Lentivirus, adeno‐associated virus, and adenoviruses are classical vectors in gene therapy.^[^ ^50^, ^51^ ^]^ Lentivirus or other vector‐mediated *PRDM16* gene delivery is a promising approach for attenuating organ aging. Screening for compounds that enhance PRDM16 transcription or protein stability is also an option. The advantage of compounds lies in their potential for oral bioavailability, tunable dosing, and a potentially more favorable safety profile compared to gene therapies.

Collectively, our study demonstrates, to our knowledge, for the first time that the loss of PRDM16 contributes to cellular senescence and multi‐organ aging. Further translational investigations targeting PRDM16 hold promise for developing potential therapeutic approaches to address the challenges of aging and aging‐related diseases.

## 4 Experimental Section

### Human Study

The normal lung tissues surrounding lung carcinoma of 13 patients were collected to investigate the mRNA level of PRDM16. Participants granted informed consent for sample usage and publication of identifiable information. And the research was approved by the Medical Ethics Committee of Union Hospital, Tongji Medical College, Huazhong University of Science and Technology (Number: UHCT230185).

Transcriptomes of heart, brain hippocampus, lung, liver and stomach were obtained from ADEIP platform (http://geneyun.net/ADEIP/).^[^ ^22^ ^]^ Tests for linear trend were conducted between age and *PRDM16* mRNA level with the use of the median value of age group as a continuous variable. Statistical analyses were conducted using R software Version 4.3.2.

Transcriptomes of 71 normal kidney samples were obtained from eligible study in Nephroseq (University of Michigan, Ann Arbor, MI).^[^ ^21^ ^]^

### Animals

The animal research protocol underwent review and approval by the Experimental Animal Ethics Committee of Tongji Medical College, Huazhong University of Science and Technology (IACUC Number: 3282) (Extended Data Ethical Approval). C57BL/6J mice (20–25 g) aged 8 weeks were purchased from Charles River Co., Ltd. The mice were housed in a temperature‐controlled environment with a 12 h light/dark cycle and were provided with free access to water and food. Animals were allocated randomly into groups. Three to six mice were used in each group according to the published papers.^[^ ^52^ ^]^

### Generation of Prdm16 Knockout Mice and Tubular‐Specific Prdm16 Knockout Mice

Global *Prdm16* knockout mice were generated by using CRISPR‐Cas9 technology at Cyagen Biosciences Inc. The exon 2 of *Prdm16* were knocked out, resulting in a frameshift mutation within the subsequent sequences. For genotyping of *Prdm16* deletion mice, PCR was performed on DNA extracted from mouse tails. The *Prdm16* deletion mutant allele (436 bp) was detected by the primers 5′‐ GGAGGTGTAGCAGGATGGTTC‐3′ (forward) and 5′‐ AACTACCACTGGAAACCAGCC‐3′ (reverse). The WT allele (557 bp) was detected by the primers 5′‐ AACATCTTGTGCCTCCTGCTG‐3′ (forward) and 5′‐ AACTACCACTGGAAACCAGCC‐3′ (reverse).

*Prdm16^flox/flox^* mice (C57BL/6J) were generated by standard homologous recombination at Shanghai Southern Model Biotechnology Development Co., Ltd. (Shanghai, China). *Prdm16* exon 9 was flanked by *loxP* sequences in the *Prdm16^flox/flox^* mice, which were bred with *Ksp‐Cre* mice to generate tubular‐specific *Prdm16* knockout mice (*Ksp‐Cre/Prdm16^flox/flox^* mice). Littermates lacking *Ksp‐Cre* expression served as controls (*Prdm16^flox/flox^* mice). The transgenic mice breeding was carried out by Shulaibao Biotechnology Co., Ltd. (Wuhan, Hubei, China). Genotyping was conducted with the DNA extracted from tails of transgenic mice. The *Prdm16^flox/flox^* mice mutant allele (379 bp) and WT allele (321 bp) was detected by the primers 5′‐ CCCACAGTGACACACCCTAC ‐3′ (forward) and 5′‐ GAGATCACGAGGAACCCCAC ‐3′ (reverse). The *Ksp‐Cre/Prdm16^flox/flox^* mice mutant allele (420 bp) was detected by the primers 5′‐ GCAGATCTGGCTCTCCAAAG ‐3′ (forward) and 5′‐ AGGCAAATTTTGGTGTACGG ‐3′ (reverse).

### Irradiation Induced Accelerated Aging Mouse Model

8‐week‐old *Ksp‐Cre/Prdm16^flox/flox^* and *Prdm16^flox/flox^* mice were anesthetized and exposed to a single dose of 10 Gy radiation using a 6‐MV X‐ray (Trilogy System Linear Accelerator, Varian Medical Systems) with a 1 cm irradiation field. The X‐ray only covered the cross‐sectional area of both kidneys. The control mice, housed under identical conditions, did not receive irradiation. 2 h months after exposure, the kidney tissues were collected for further analysis.

### IeR and Unilateral Ischemia Reperfusion Injury Mouse Model

8‐week‐old *Ksp‐Cre/Prdm16^flox/flox^* and *Prdm16^flox/flox^* mice underwent radiation exposure as mentioned above. 2 months after IR, the left renal pedicle was clipped for 40 min with vascular clamp, and body temperature was maintained at 35 °C using a constant temperature metal bath. The right kidney was surgically excised 10 days after UIRI, and mice were sacrificed after 24 h. The kidney tissues and serum were collected.

### Aging Mouse Model with PRDM16 Lentivirus (PR‐LV)

C57BL/6J mice were raised to 23 months old. Negative control (NC) lentivirus or PRDM16 overexpressing lentivirus (5 × 10^6^ IU) was dissolved in 100 µL of cold sterile PBS and injected slowly into the inferior and superior poles of the kidney using 8 mm insulin needles. After 1 month, the kidney tissues were collected for further analysis.

### IR Mouse Model with GSTM1 Lentivirus (GSTM1‐LV)

8‐week‐old *Prdm16^flox/flox^* and *Ksp‐Cre/Prdm16^flox/flox^* mice underwent radiation exposure as mentioned above. 2 months after IR, NC lentivirus or GSTM1 overexpressing lentivirus (5 × 10^6^ IU) was dissolved in 100 µL of cold sterile PBS and injected slowly into the inferior and superior poles of the kidney using 8 mm insulin needles. 1 month after lentivirus injection, the kidney tissues were collected for further analysis.

### Serum Biochemistry Tests

Serum parameters including AST, ALT, albumin and glucose were detected using an automatic biochemical analyzer. Serum creatinine was determined using a Creatinine Assay Kit (DICT‐500, Bioassay Systems). And the serum albumin to creatinine ratio (ACR) was calculated.

### Detection of Reactive Oxygen Species

The ROS levels of kidney tissues and HK‐2 cells were detected using the Dihydroethidium (DHE) Fluorescent Probe (S0063, Beyotime). In summary, the kidney sections or HK‐2 cells were incubated with DHE fluorescent probe for 45–60 min at 37 °C, followed by nuclear staining with DAPI. Microscope images were then captured.

### Transmission Electron Microscopy

Renal cortical tissue samples were cut into 1 cm^3^ pieces and fixed in 2.5% glutaraldehyde. After fixation, samples were dehydrated in 3%, 5%, 70%, 90% and 100% acetone. Then the samples were embedded in ethoxy resin. The samples were handled, and TEM was performed by the Electron Microscopic Lab of Department of Nephrology, Union Hospital, Tongji Medical College, Huazhong University of Science and Technology.

### Cell Culture and Treatments

Human proximal tubule epithelial (HK‐2) line, and human embryonic kidney 293T (HEK‐293T) cell line were obtained from American Type Culture Collection (ATCC). HK‐2 cells were cultured in DMEM/F12 medium (Gibico) containing 10% FBS (VivaCell) and 1% penicillin/streptomycin (NCM) in a 37 °C incubator with 5% CO_2_. HEK‐293T cells were cultured in DMEM medium (Gibico) containing 10% FBS (VivaCell). For IR induced senescence research, HK‐2 cells were exposed to a single dose of 8 Gy radiation. Cells were cultured for 7 days, and the medium was changed every 2 days. For D‐gal induced senescence research, HK‐2 cells were cultured in medium containing 1% FBS and 10 mg mL^−1^ D‐gal (G5388, Sigma) for 72 h.

Bronchial epithelium transformed with Ad12‐SV40 2B (Beas‐2B) line was obtained from ATCC. Beas‐2B cells were cultured in DMEM medium (Gibico) containing 10% FBS (VivaCell) and 1% penicillin/streptomycin (NCM) in a 37 °C incubator with 5% CO_2_. For BLM induced senescence research, Beas‐2B cells were treated with 6 µg mL^−1^ BLM (HY‐17565, MCE) for 48 h.

Rat embryonic cardiomyocyte (H9C2) line was obtained from American Type Culture Collection (ATCC). H9C2 cells were cultured in DMEM medium (Gibico) containing 10% FBS (VivaCell) and 1% penicillin/streptomycin (NCM) in a 37 °C incubator with 5% CO_2_. For DOX induced senescence research, H9C2 cells were treated with 1 µM DOX (D107159, Aladdin) for 24 h.

### Lentivirus‐Mediated Gene Expression In Vitro

HK‐2 cells were cultured in 6‐well plates at a density of 20–30%. Then cells were treated with co‐transfection reagents and lentivirus at a multiplicity of infection (MOI) of 10. The medium was replaced after 12 h of transfection. At 72 h post‐transfection, GFP fluorescence was observed, and 2 µg mL^−1^ puromycin (ST551, Beyotime) was added in the medium to eliminate non‐transfected cells.

### siRNA‐Mediated Knockdown

HK‐2 Cells were incubated in antibiotic‐free medium. The Lipofectamine 2000 reagent (Invitrogen, Carlsbad, CA) was utilized to deliver short interfering RNA (siRNA) specific to the target genes or scramble control (also known as negative control siRNA) into the cells according to the manufacturer's instructions.

### RNA Isolation and Quantitative Real‐Time PCR

RNA was extracted from tissues or cells using RNAiso Plus reagent (9109, Takara) following the recommended procedures. cDNA was synthesized using the HiScript III RT SuperMix for qPCR (+gDNA wiper) (R323, Vazyme). Quantitative real‐time PCR was conducted using ChamQ SYBR qPCR Master Mix (R311, Vazyme) in the QuantStudio 1 Real‐Time PCR System (Thermo Fischer Scientific, USA). The primers utilized for detecting target mRNA were listed in Extended Data Table S3, Supporting Information. Analysis of gene expression was performed employing the 2^−(ΔΔCt)^ method.

### Western Blotting

Tissues or cells were lysed using ice‐cold RIPA lysis buffer (Beyotime) containing protease inhibitor cocktail (HY‐K0010, MCE), PMSF (AR1192, BOSTER) and phosphatase inhibitors (AR1183, BOSTER). The protein concentration was determined by the BCA Protein Assay Kit (23 227, Thermo Fischer Scientific). After boiling for 10 min at 95 °C in SDS protein‐loading buffer (AR1112, BOSTER), equal amounts of protein were separated through SDS‐PAGE and subsequently transferred onto PVDF membranes (Millipore). The membranes were blocked with 5% non‐fat milk for 1 h, and then incubated with primary antibodies at 4 °C overnight. Subsequently, the membranes were incubated with either horseradish peroxidase‐conjugated anti‐rabbit antibody (BA1055, BOSTER) or anti‐mouse antibody (BA1050, BOSTER) for 1 h at 25 °C. Resulting blots were visualized using ECL Western Blotting Substrate (BMU102‐CN, Abbkine) and a BioSpectrum Imaging System (UVP, USA) following the manufacturers’ instructions. Image analysis was performed utilizing ImageJ software (NIH, USA). The relevant antibodies were listed in Extended Data Table S2, Supporting Information.

### Chromatin Immunoprecipitation Analysis

The interaction between proteins and DNA was investigated via ChIP‐PCR using a ChIP assay kit (P2078, Beyotime). In summary, cells and ground kidney tissues were collected and crosslinked with 1% formaldehyde for 10 min at room temperature. Following sonication, primary antibody‐mediated immunoprecipitation was carried out. The complex obtained from immunoprecipitation was washed, and DNA was extracted and purified using the DNA Purification Kit (D0033, Beyotime). The purified samples underwent PCR amplification and DNA agarose gel electrophoresis was performed. The primer sequences are listed in Extended Data Table S3, Supporting Information.

### Histological Analysis

The 4% paraformaldehyde fixed tissue samples were utilized to prepare paraffin‐embedded sections (3 µm) which were subsequently stained with H&E for histological analysis. Masson's trichrome staining (MTS) was carried out to assess the degree of fibrosis. Immunohistochemical analyses were conducted to detect protein expression in the tissues. In brief, paraffin‐embedded sections were incubated in specified primary antibodies (Extended Data Table S2, Supporting Information) and then visualized using DAB kit. Sections were further stained with hematoxylin. The stained sections were observed under a microscope (Ni‐E, Nikon).

### Immunofluorescence

Cells seeded in coverslips were fixed in 4% paraformaldehyde for 20 min and then treated with 0.3% Triton‐X 100 for 15 min for permeabilization. Paraffin‐embedded sections were deparaffinized and rehydrated, followed by antigen unmasking and permeabilization procedures. Coverslips or sections were then incubated with 10% donkey serum before overnight incubation with primary antibodies or WGA dye (Extended Data Table S2, Supporting Information). Subsequently, coverslips or sections were incubated with secondary antibodies for 1 h followed by nuclear staining with DAPI. Microscope images were then captured.

### SA‐β‐Gal Staining

The SA‐β‐gal activities of kidney frozen sections (5 µm) and cells seeded in coverslips were detected as per the guidelines of manufacturer (C0602, Beyotime). Sections were further stained with hematoxylin. Images were captured and then the SA‐β‐gal positive cell ratio or area ratio were calculated via ImageJ software.

### Single‐Cell RNA Sequencing

Fresh renal cortex tissues were excised and stored in MACS Tissue Storage Solution (Miltenyi Biotec) before further processing. In summary, the samples were washed with PBS and minced into small fragments of ≈1 mm^3^ while maintained on ice. Subsequently, the minced samples underwent enzymatic digestion utilizing a combination of 1 mg mL^−1^ Collagenase I (Gibco), 1 mg mL^−1^ Collagenase II (Gibco), 60 U mL^−1^ Hyaluronidase (Sigma), 10 U mL^−1^ Liberase (Roche) and 0.02 mg mL^−1^ DNase I (Roche) for 90 min at 37 °C with agitation. Following digestion, the samples were filtered through 100 µm and 40 µm cell strainers and centrifuged at 300 g for 5 min. After removing the supernatant, the resulting cell pellets were resuspended in red blood cell lysis buffer (Gibco) to facilitate the lysis of red blood cells. Finally, the cell pellets were washed with Dulbecco's phosphate‐buffered saline (DPBS) containing sample buffer, and then resuspended, stained and counted in the same buffer.

The BD Rhapsody system was utilized to capture the transcriptomic information of the single cells derived from renal cortex. Single‐cell capture was achieved through the random distribution of a suspension containing single cells into over 200 000 microwells via a limited dilution method. Beads embedded with oligonucleotide barcodes were added to saturation, ensuring that each bead in the microwell was paired with a single cell. The cells underwent lysis within the microwell, allowing for the hybridization of mRNA to the barcoded capture oligos present on the beads. Subsequently, the beads were collected into a single tube for the processes of reverse transcription and ExoI digestion. During the synthesis of cDNA, each cDNA molecule was labeled at the 5′ end (corresponding to the 3′ end of its mRNA transcript) with a unique molecular identifier (UMI) and a barcode designating the cell origin of it. Whole transcriptome libraries were constructed by employing the BD Rhapsody single‐cell whole‐transcriptome amplification (WTA) workflow, which includes random priming and extension (RPE), RPE amplification PCR, and WTA index PCR. The libraries underwent quantification using a High Sensitivity D1000 ScreenTape (Agilent) along with High Sensitivity D1000 Reagents (Agilent) on a 4150 TapeStation System (Agilent) and assessed via the Qubit High Sensitivity DNA assay (Thermo Fisher Scientific). Sequencing was carried out using an Illumina sequencer (Illumina, San Diego, CA) in a paired‐end run of 150 bp.

### Single‐Cell RNA Sequencing Analysis

The BD Rhapsody WTA Analysis Pipeline (version 1.8) was employed to generate sequencing libraries from single cell transcriptomes, notably without specifying a targeted panel. Following the sequencing process, the analysis pipeline utilized FASTQ files, a reference genome file mm10, and a transcriptome annotation file GENCODE vM23/Ensembl 98 to perform sequence alignment. This pipeline produced a UMI count matrix that was subsequently processed with the Python package Scanpy (version 1.8) for further analysis.^[^ ^53^ ^]^ To eliminate low‐quality cells, cells with UMI or gene counts below the specified threshold (500 ≤ UMI ≤ 60 000, 200 ≤ gene counts ≤ 8000) were excluded. After this initial filtering process, a visual inspection was conducted to assess the distribution of cells based on the fraction of mitochondrial genes expressed. Following the application of these quality control criteria, a total of 15 833 single cells from the renal cortex of 9‐month‐old WT mice and 18 232 single cells from the renal cortex of 9‐month‐old *Prdm16* KO mice were retained for downstream analysis. Library size normalization was carried out using the pp.normalize_total function in Scanpy to derive normalized counts. Specifically, the global‐scaling normalization method adjusted the gene expression data for each cell according to the total expression, multiplied by a scaling factor (default is 10 000), and applied the pp.log1p function for log transformation.

Top variable genes across single cells were determined using the methodology described by Macosko et al.^[^ ^54^ ^]^ The selection of the most variable genes was carried out through the pp.highly_variable_genes function available in Scanpy. Principal component analysis (PCA) was conducted to reduce the dimension of dataset via utilizing the tl.pca function in Scanpy. The graph of cell clustering based on gene expression profiles was performed by applying the pp.neighbors function in Scanpy. Visualization of cells was carried out using both 2D and 3D Uniform Manifold Approximation and Projection (UMAP) methods, employing the tl.umap function in Scanpy. The FindAllMarkers function (test.use = wilcox) in Seurat was utilized to identify marker genes within each cluster.^[^ ^55^ ^]^ For any specified cluster, FindAllMarkers identified positive markers in relation to all other cells, with adjusted *p* value < 0.05 and |log2foldchange| > 0.26 established as the criteria for significant marker genes. The ClusterProfiler package of R software was employed to conduct functional enrichment analysis on the marker genes.^[^ ^56^ ^]^ The marker genes corresponding to each cluster were mapped to established sources of functional pathways, and the hypergeometric distribution was utilized to determine whether these biological processes were over‐represented, encompassing pathways from Gene Ontology (GO), KEGG, and others. The sequencing and bioinformatics analysis were performed by Genechem Co., Ltd. (Shanghai, China).

### RNA‐Seq Analysis

Total RNA extraction was performed on irradiated HK‐2 cells transfected with negative control lentivirus or PRDM16 overexpression lentivirus, followed by RNA‐seq analysis utilizing the MGI platform. For RNA quality assessment, a NanoDrop 2000 spectrophotometer (NanoDrop Technologies, Wilmington, DE, USA) and a Bioanalyzer 2100 system (Agilent Technologies, CA, USA) were employed. After purification with Oligo(dT)‐attached magnetic beads, mRNA was fragmented into smaller segments in a fragment buffer. Thereafter, the first strand of cDNA was synthesized via random hexamer‐primed reverse transcription, and the second strand was generated and subsequently purified with AMPure XP Beads. The construction of the cDNA library involved various steps including repair, PCR amplification, and purification. For quantification, both Qubit 2.0 and Agilent 2100 bioanalyzer were utilized. Sequencing was conducted by BGI Hong Kong on the MGI DNBseq T7 platform, yielding 150 bp paired‐end reads. Raw reads from the RNA‐seq libraries were processed to eliminate those exhibiting low quality or containing adapters. After the filtering process, statistical analysis was conducted with R software. The library construction, sequencing, and subsequent analysis were managed by Wuhan Generead Biotechnology Co. Ltd (Wuhan, China).

### Serum Cytokine Array

Fresh serum samples of mice were collected to detect senescence‐associated secretory phenotype. Aliquots (50 µl) of serum samples were tested using the ABplex Custom Panel Assay Kit.

### Echocardiography

Transthoracic echocardiography was conducted on mice using a VEVO‐1100 ultrasound system (VisualSonics) to evaluate cardiac function. Mice were anesthetized with 1% isoflurane inhalation to maintain heart rates between 450 and 550 beats min^−1^. Images were captured from a parasternal long‐axis perspective, as well as a short‐axis view at the level of the papillary muscle, and 2D‐guided M‐mode images were recorded. Ejection fraction (EF), fractional shortening (FS), cardiac output, and stroke volume were determined by averaging measurements from at least five consecutive cardiac cycles for each sample.

### Forelimb Grip Strength Test

To evaluate the strength of limbs in mice, an automatic grip strength meter was utilized. The procedure involved suspending the mouse by its tail and pulling it gently backward, prompting the mouse to grasp onto a grid. While being pulled away from the apparatus, the maximum resistance force when the mouse released its grip on the grid was measured.

### Rotarod Test

The rotating rod test was conducted utilizing a rotating device (YLS‐4C, Yiyan). Prior to the test, the mouse was acclimatized to stay on the rod for 2 min. Subsequently, each mouse was positioned on a device rotating at a consistent velocity ranging from 5 to 40 rpm for 5 min and underwent 3 trials with a 30‐min gap between each trial. The mice were promptly returned to cages after each trial, and the time taken for them to fall was automatically documented. Mice that remained on the device for over 5 min were noted as 5 min and then removed from the device. The rotating rod was cleaned with 75% alcohol before the next trial. The average fall time from the 3 trials reflects the motor ability of the mice.

### Morris Water Maze

The MWM test comprises acquisition training and a probe trial. During the acquisition training phase, mice were trained to search the platform hidden 1 cm below the water, guided by constant cues outside the pool. In each trial, the mice were allowed to locate the hidden platform for 60 s. If the mice failed to find the platform, they would receive guidance to reach and remain on the hidden platform for 15 s. The acquisition training consisted of four trials per day over 6 consecutive days. On the seventh day, a probe trial was conducted to evaluate the ability of memory. The platform was removed, allowing the mice to swim for 60 s. Their behaviors were recorded and analyzed using the SuperMaze software. And the water temperature was maintained at 26–28 °C.

### Glutathione‐S‐Transferase Activity and GSH/GSSG Ratio

The GST activity of kidney tissues or HK‐2 cells were measured using GST Activity Assay Kit (E‐BC‐K278‐S, Elabscience) according to the instruction. And the total protein concentration was determined by the BCA Protein Assay Kit (23 227, Thermo Fischer Scientific). The GSH and GSSG content of kidney tissues or HK‐2 cells were detected by GSH and GSSG Assay Kit (S0053, Beyotime). And the GSH/GSSG ratio was calculated.

### Luciferase Reporter Assay

The GSTM1 promoter was inserted into the firefly luciferase reporter plasmid. HEK‐293T cells were co‐transfected with firefly luciferase reporter plasmid, either negative control siRNA or PRDM16 siRNA, using Lipofectamine 2000 reagent. To normalize the transfection efficiency, Renilla reniformis luciferase reporter plasmid (Promega, USA) was also transfected. The luciferase activity was measured at 48 h post‐transfection by using the Dual‐Luciferase Reporter Kit (Promega, USA).

### Statistical Analysis

All data were presented as mean ± SEM. Two‐tailed Student's unpaired *t*‐test analysis was used for two‐groups comparison and one‐way ANOVA followed by Tukey's post‐test was used for multiple‐group comparison. Spearman's correlation analysis was used to evaluate the association between the mRNA level of *PRDM16*, age and the mRNA level of *CDKN1A*. Test for linear trend was used for the relationship between the level of *PRDM16* and age. All statistical analysis were two‐sided and *p* < 0.05 was considered significant. Single‐cell RNA sequencing data was analyzed using Python 3 and R 4.3.2, and the other statistical analysis were performed using GraphPad Prism 9.0 or R 4.3.2.

## Conflict of Interest

The authors declare no conflict of interest.

## Author Contributions

Q.Y. and Y.T.Z. contributed equally to this work. Q.Y. and C.Z. designed the study; Y.T.Z., M.C.H., and H.S. collected and analyzed the clinical data; Q.Y., Y.T.Z., B.T., and Y.R.X. performed animal models; Y.T.Z. performed in vitro experiments; Q.Y. and Y.T.Z. prepared figures and tables; Q.Y. and Y.T.Z. wrote the paper; C.Z., Y.H.L., and Q.Y. revised and approved the final version of the manuscript.

## Supporting information



Supporting Information

## Data Availability

The bulk RNA‐Seq data from irradiated HK‐2 cells transfected with negative control lentivirus or PRDM16 overexpression lentivirus was deposited in the NCBI's GEO database (GSE275768). The scRNA‐seq data from the renal cortex of 9‐month‐old WT and global *Prdm16* KO mice was deposited in the NCBI's GEO database (GSE307024). The materials used in this study should be directed to and will be fulfilled by the Lead Contac, Doctor Chun Zhang: drzhangchun@hust.edu.cn.
