# Supplementary material for: PRDM16 Reduces Cellular Senescence by Upregulating GSTM1
Source: Adv Sci (Weinh). 2025 Sep 14;12(45):e01233. doi: 10.1002/advs.202501233 (PMC12677672; doi:10.1002/advs.202501233)

**Supplementary materials**

**PRDM16 Reduces Cellular Senescence by Upregulating GSTM1**


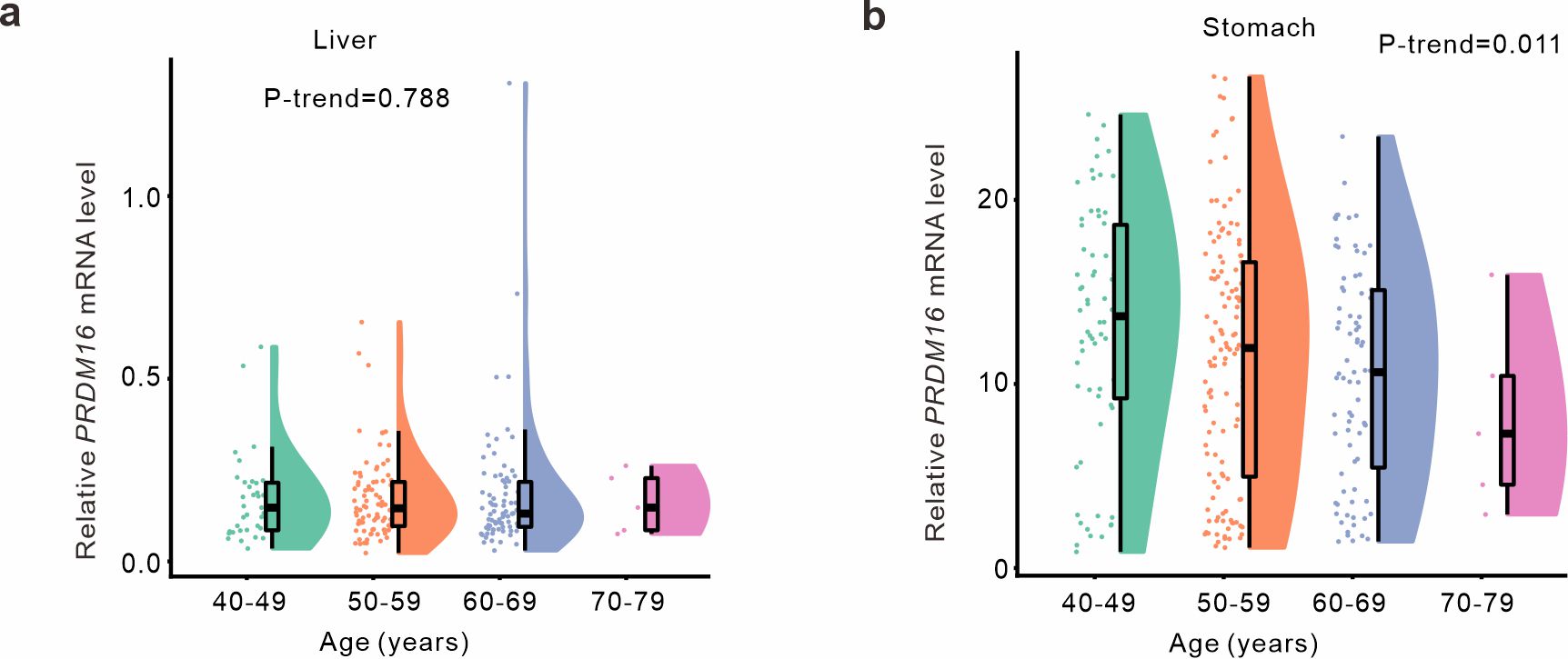


**Extended Data Figure 1. The association between age and *PRDM16* mRNA level in liver and stomach.**

**(****a and b)** Tests for linear trend were conducted between age and *PRDM16* mRNA level in liver **(a)** (n=202) and stomach **(b)** (n=276) based on transcriptomes from ADEIP.

**
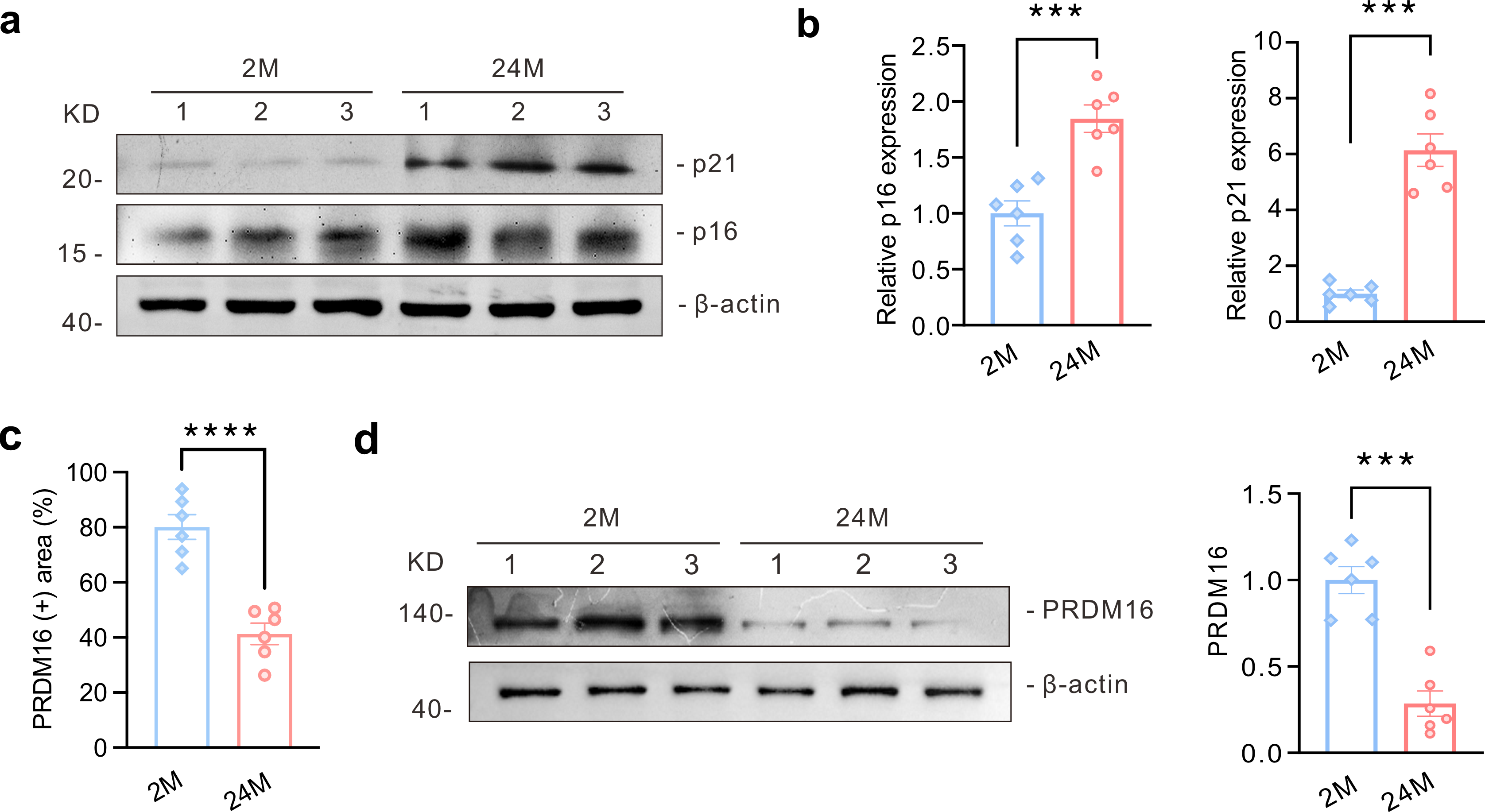
**

**Extended Data Figure 2. The protein levels of senescence markers and PRDM16 in the kidneys of aged mice.**

**(a and b)** A representative western blot **(a)** and quantification **(b)** of senescence markers in the kidney cortex of mice at 2 months old and 24 months old (n=6). Numbers (1-3) represent different animals in a given group. **(c)** The percentage of PRDM16 positive area was calculated (n=6). **(d)** A representative western blot and quantification of PRDM16 in the kidney cortex (n=6). Numbers (1-3) represent different animals in a given group. Data are mean ± SEM. ****P* < 0.001, *****P* < 0.0001. Two-tailed Student’s unpaired t test analysis (b-d).


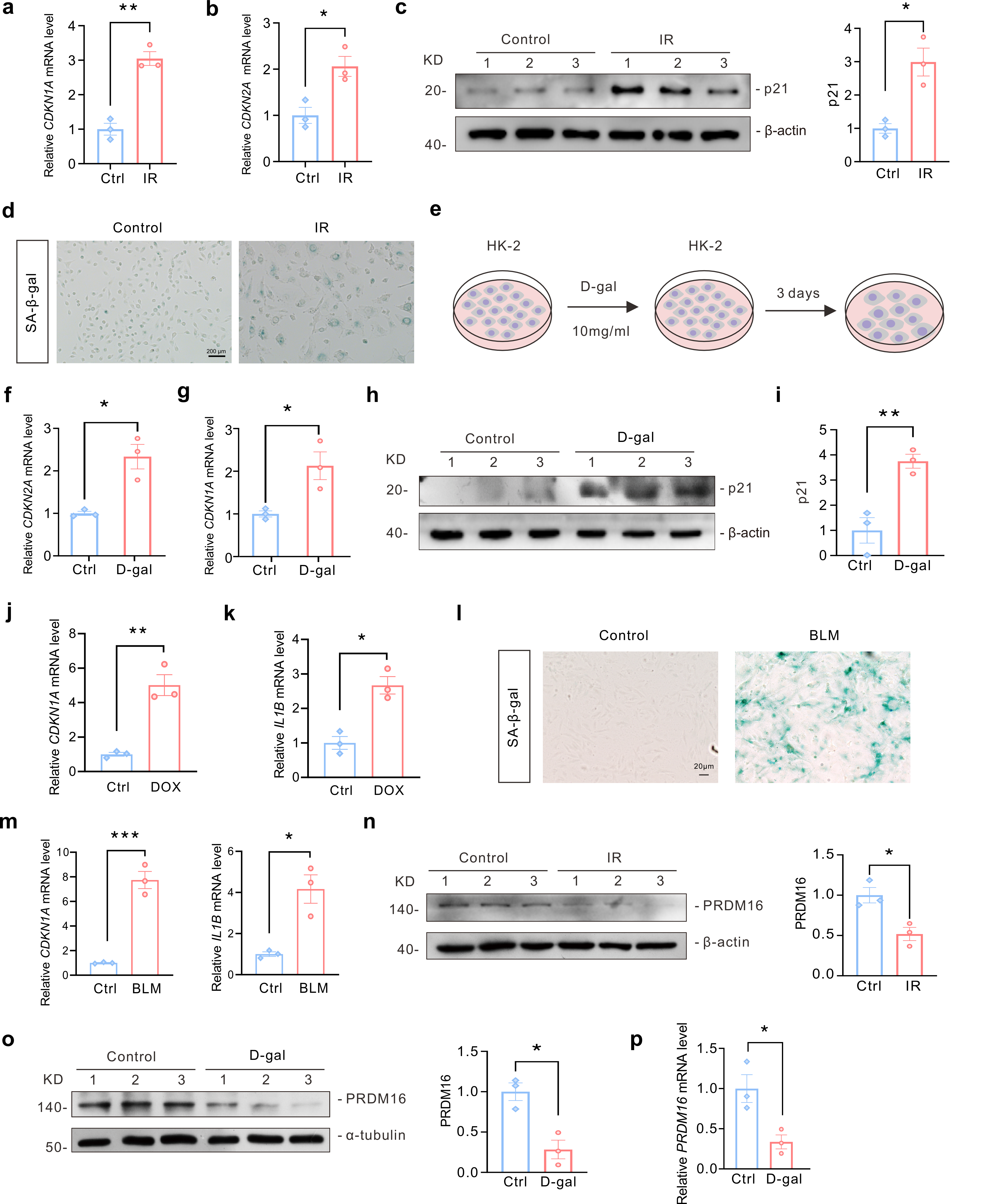


**Extended Data Figure 3. PRDM16 was downregulated in senescent cells.**

**(a and b)** qPCR analysis of *CDKN1A* **(a)** and *CDKN2A* **(b)** in irradiated HK-2 cells (n=3). **(c)** A representative western blot **(c)** and quantification of p21 in irradiated HK-2 cells (n=3). Numbers (1-3) represent different wells of cells in a given group. **(d)** Representative images of SA-β-gal staining in irradiated HK-2 cells. Scar bar: 200 μm. **(e)** Diagram detailing D-galactose (D-gal) induced cellular senescence in HK-2 cells. **(f and g)** qPCR analysis of *CDKN2A* **(f)** and *CDKN1A* **(g)** in D-gal treated HK-2 cells (n=3). **(h and i)** A representative western blot **(h)** and quantification **(i)** of p21 in D-gal treated HK-2 cells (n=3). Numbers (1-3) represent different wells of cells in a given group. **(j)** qPCR analysis of *CDKN1A* in DOX treated H9C2 cells (n=3). **(k)** qPCR analysis of *IL1B* in DOX treated H9C2 cells (n=3). **(l)** Representative images of SA-β-gal staining in BLM treated Beas-2B cells. Scar bar: 20 μm. **(m)** qPCR analysis of *CDKN1A* and *IL1B* in BLM treated Beas-2B cells (n=3). **(n)** A representative western blot and quantification of PRDM16 in irradiated HK-2 cells (n=3). Numbers (1-3) represent different wells of cells in a given group. **(o)** A representative western blot and quantification of PRDM16 in D-gal treated HK-2 cells (n=3). Numbers (1-3) represent different wells of cells in a given group. **(p)** qPCR analysis of *PRDM16* in D-gal treated HK-2 cells (n=3). Data are mean ± SEM. **P* < 0.05, ***P* < 0.01 and ****P* < 0.001. Two-tailed Student’s unpaired t test analysis (a-c, f, g, i-k, m-p).

**
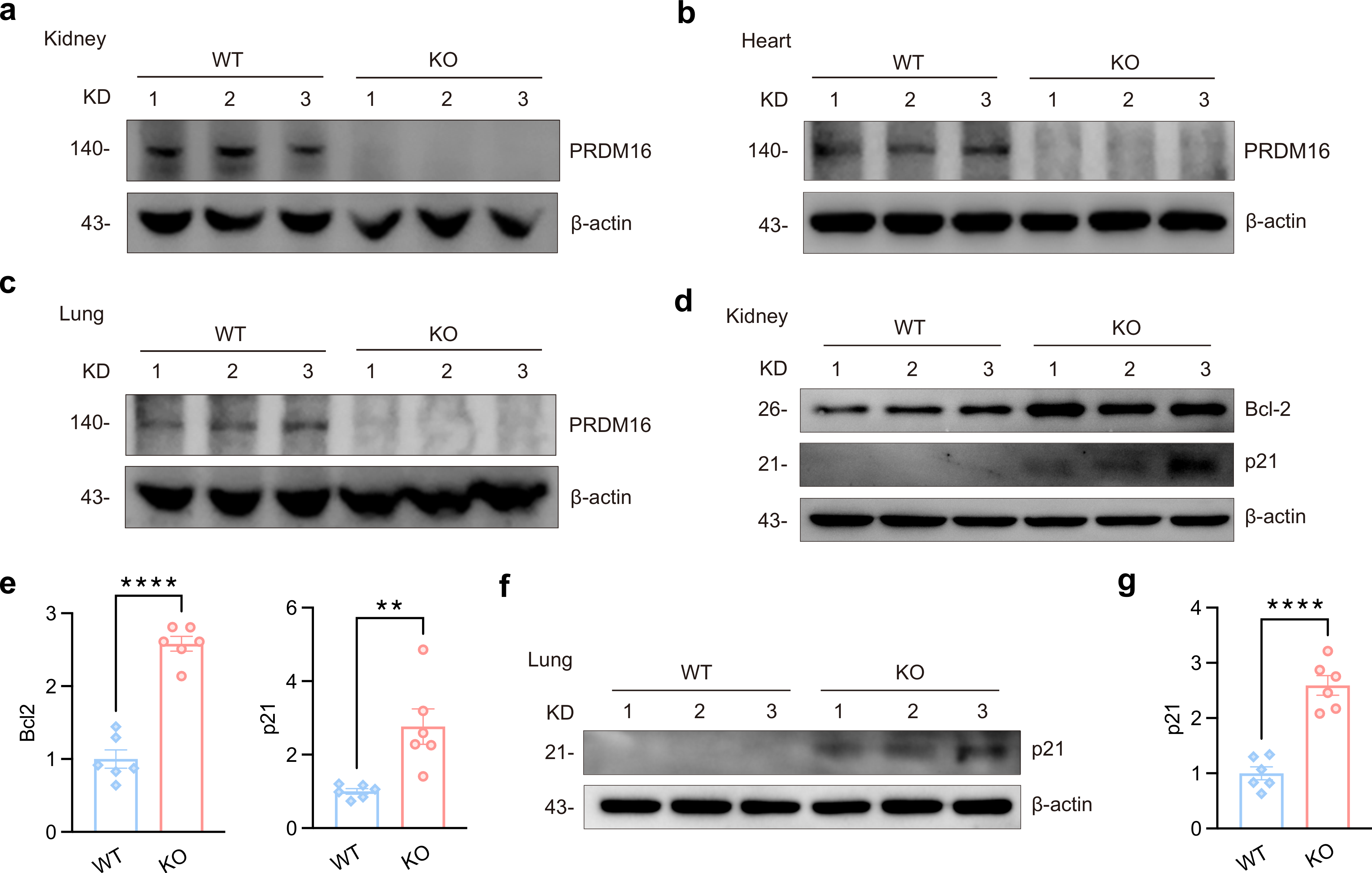
**

**Extended Data Figure 4. Global knockout of *Prdm16* contributed to senescence in multiple organs.**

**(a-c)** Representative western blots of PRDM16 in the kidney **(a)**, heart **(b)** and lung **(c)** of wide type (WT) and *Prdm16* knockout (KO) mice (n=3). Numbers (1-3) represent different animals in a given group. **(d and e)** A representative western blot **(d)** and quantification **(e)** of senescence markers in the kidney cortex of wide type (WT) and *Prdm16* knockout (KO) mice (n=6). Numbers (1-3) represent different animals in a given group. **(f and g)** A representative western blot **(f)** and quantification **(g)** of p21 in the lung of wide type (WT) and *Prdm16* knockout (KO) mice (n=6). Numbers (1-3) represent different animals in a given group. Data are mean ± SEM. ***P* < 0.01 and *****P* < 0.0001. Two-tailed Student’s unpaired t test analysis (e and g).


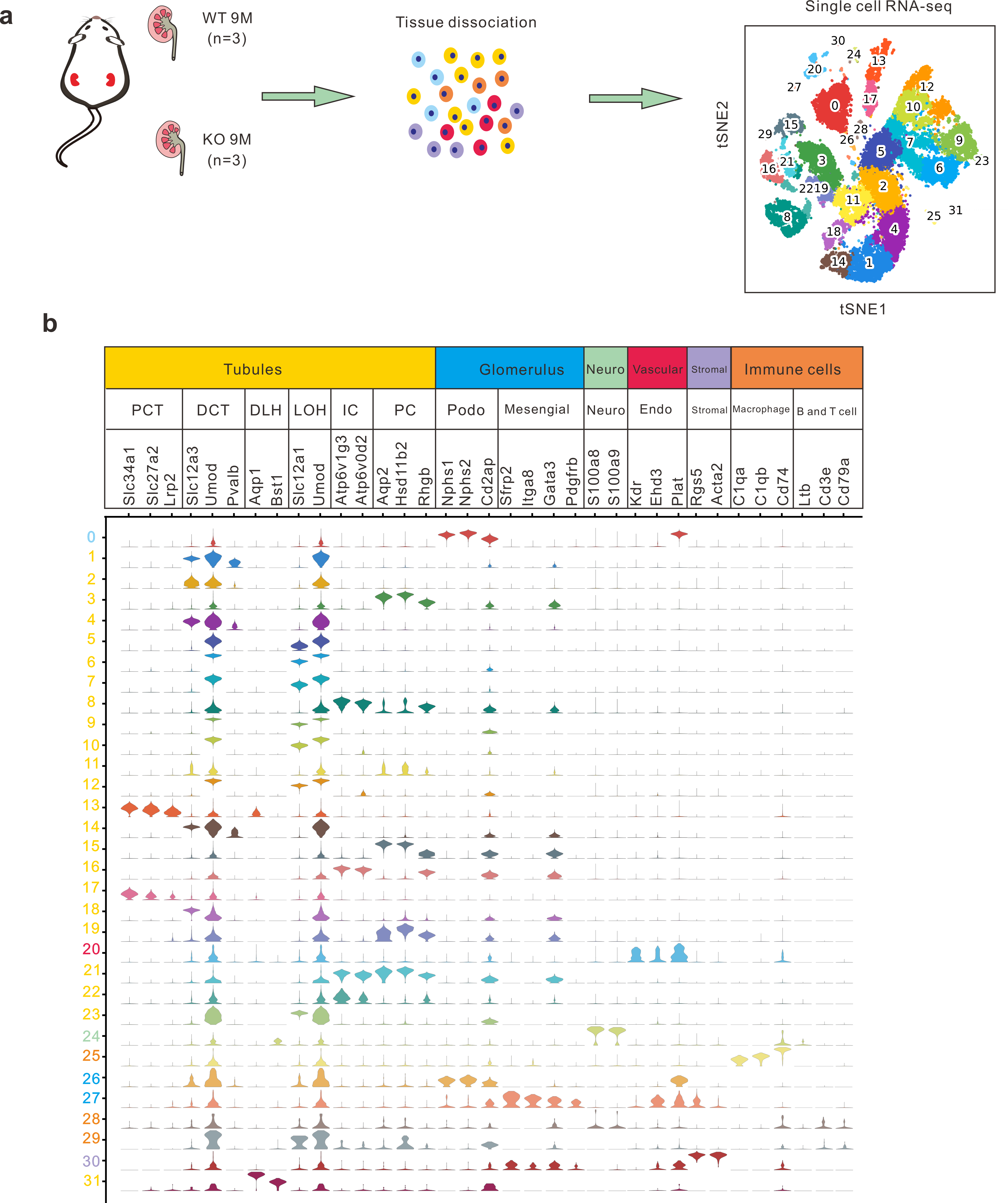


**Extended Data Figure 5. A single-cell atlas of mouse kidney revealed major cell-type identities.**

**(a)** Diagram detailing the BD Rhapsody workflow used to analyze single-cell suspensions from whole renal cortex of 9-month-old wild type (WT) and *Prdm*16 knockout (KO) mice. The plot of t-distributed stochastic neighbor embedding (tSNE) indicates unsupervised clustering of transcriptomes, uncovering 31 unique cellular clusters. (n=3). **(b)** Violin plots showing the expression level of respective selected marker genes in all cellular clusters.


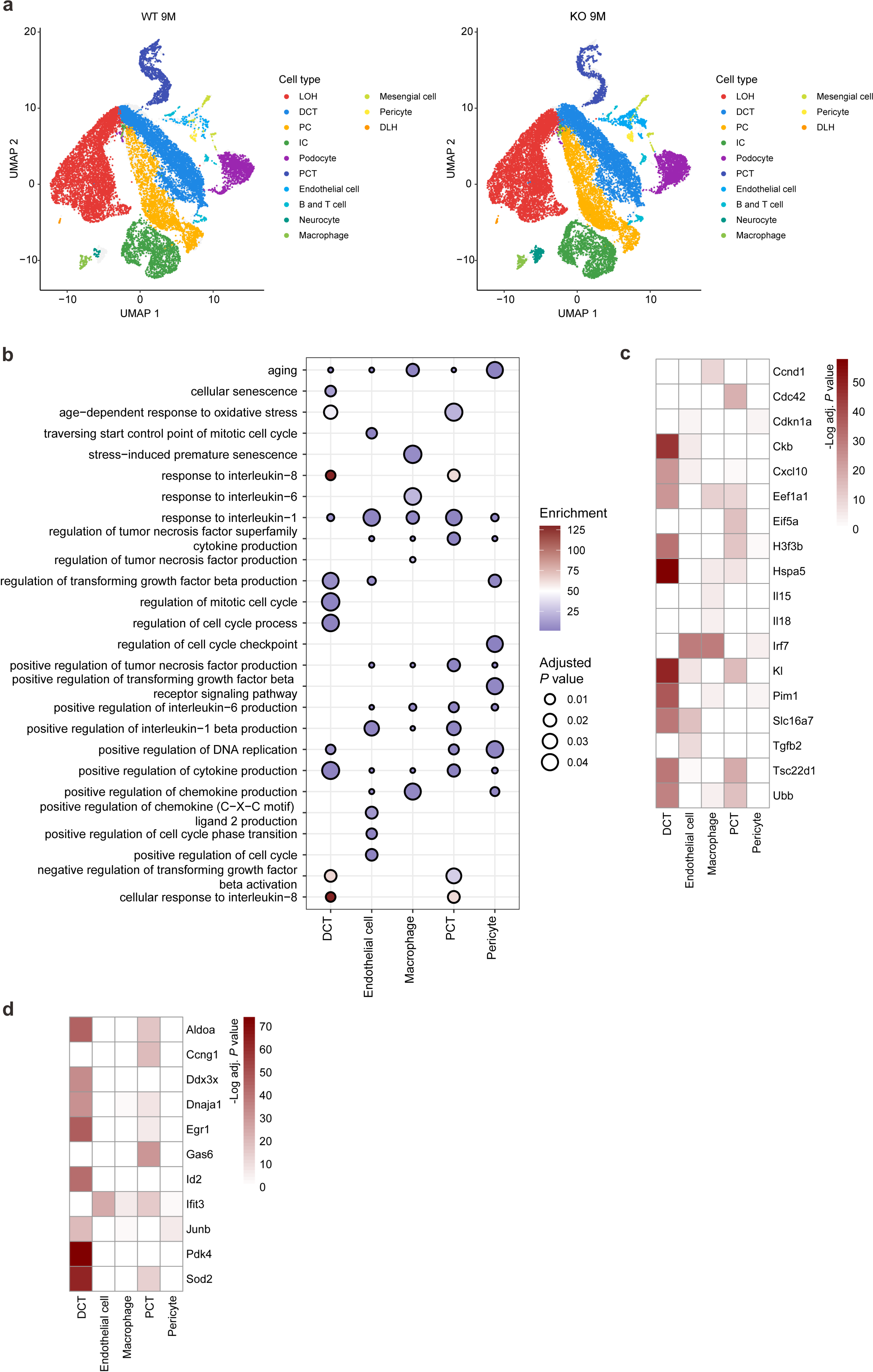


**Extended Data Figure 6. *Prdm16* deficiency affected senescence and DNA damage across different cell types.**

**(a)** UMAP plots showing cellular clusters in the renal cortex of 9-month-old wild type (WT) and *Prdm*16 knockout (KO) mice (n=3). **(b)** GO terms associated with senescence affected by *Prdm16* deficiency across different cell types (n=3). **(c)** Senescence-related genes affected by *Prdm16* deficiency across different cell types (n=3). **(d)** DNA damage related genes affected by *Prdm16* deficiency across different cell types (n=3).


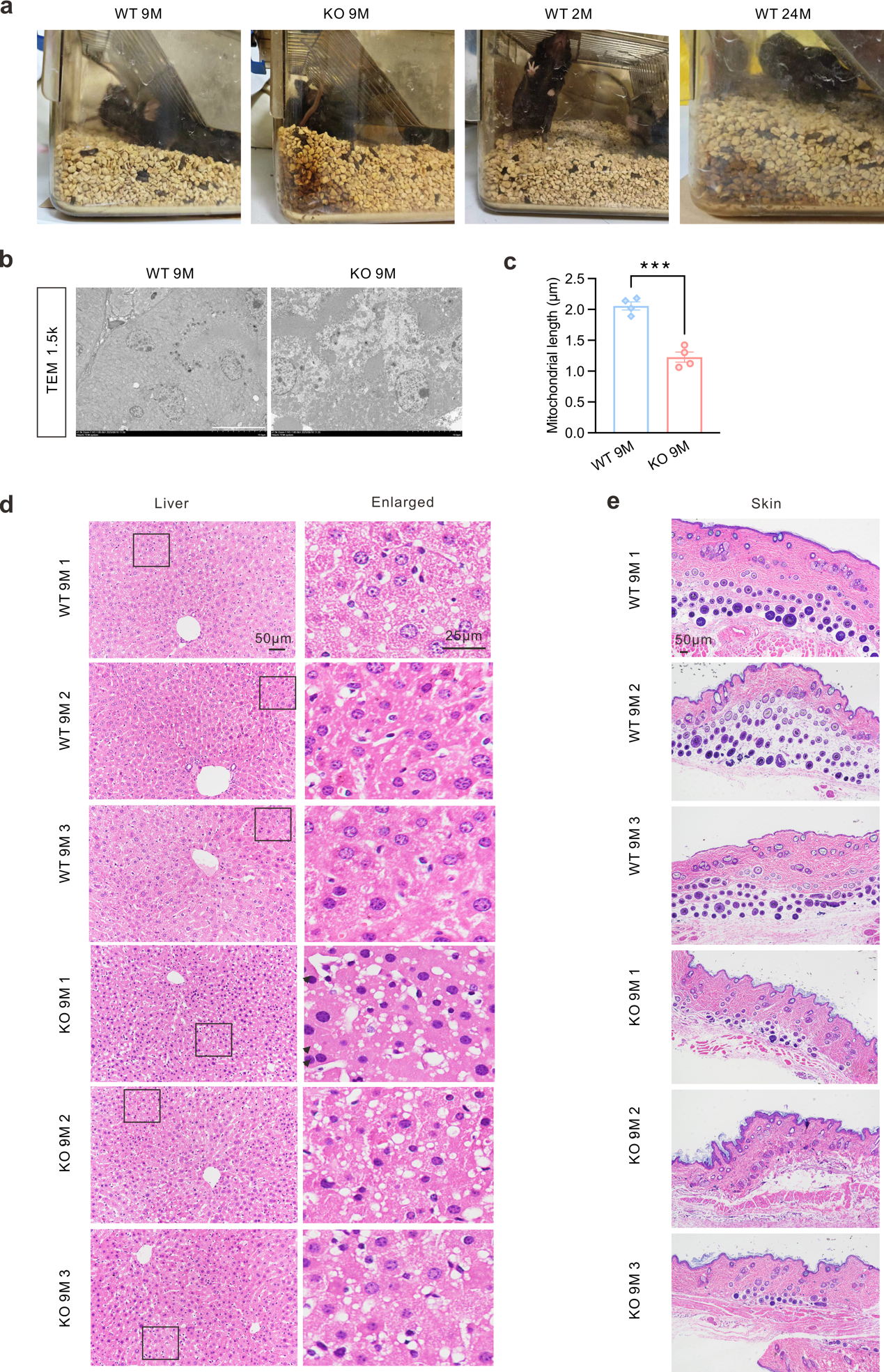


**Extended Data Figure 7. Genetic deletion of *Prdm16* contributed to liver and skin injury.**

**(a)** The bedding of 9-month-old wide type (WT) and *Prdm16* knockout (KO) mice under the same housing condition (left two panels). The bedding of 2-month-old and 24-month-old WT mice under the same housing condition (right two panels). **(b)** Representative transmission electron microscopy (TEM) images in the tubular epithelial cells of mice. Scale bar: 10 µm. **(c)** Mitochondrial length in tubular epithelial cells of mice was measured (n=4). **(d)** Representative Hematein Eosin images of liver in WT and *Prdm16* KO mice (9 months old) (n=3). Scale bar: 50 µm (left panel) and 25 µm (right panel). **(e)** Representative Hematein Eosin images of skin (n=3). Scale bar: 50 µm. Data are mean ± SEM. ****P* < 0.001. Two-tailed Student’s unpaired t test analysis (c).


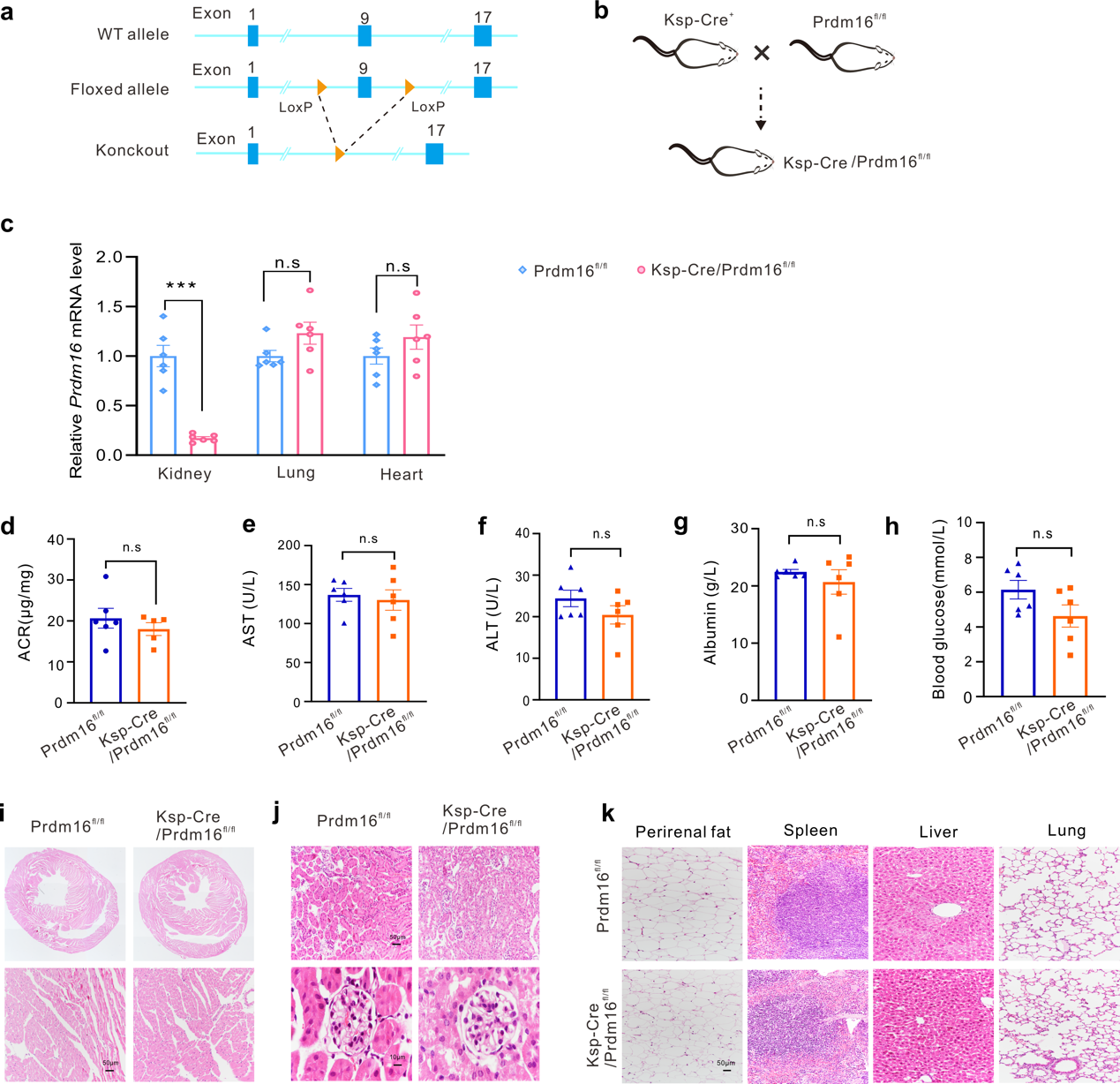


**Extended Data Figure 8. Generation of the tubular-specific *Prdm16* deletion mice.**

**(a and b)** Diagram detailing the strategy to generate *Ksp-Cre/Prdm16^fl/fl^* mice. **(c)** qPCR analysis of *Prdm16* in the kidney, lung and heart (n=6). **(d-h)** Urine albumin to creatinine ratio (ACR) **(d)**, serum AST **(e)**, serum ALT **(f)**, serum albumin **(g)** and serum glucose **(h)** were measured and calculated (n=6). **(i)** Representative Hematein Eosin images of heart. Scale bar: 50 µm. **(j)** Representative Hematein Eosin images of renal tubules (upper panel) and glomeruli (lower panel). Scale bar: 50 µm (upper panel) and 10 µm (lower panel). **(k)** Representative Hematein Eosin images of perirenal fat, spleen, liver and lung. Scale bar: 50 µm. Data are mean ± SEM. ****P* < 0.001. n.s: not significant. Two-tailed Student’s unpaired t test analysis (c-h).


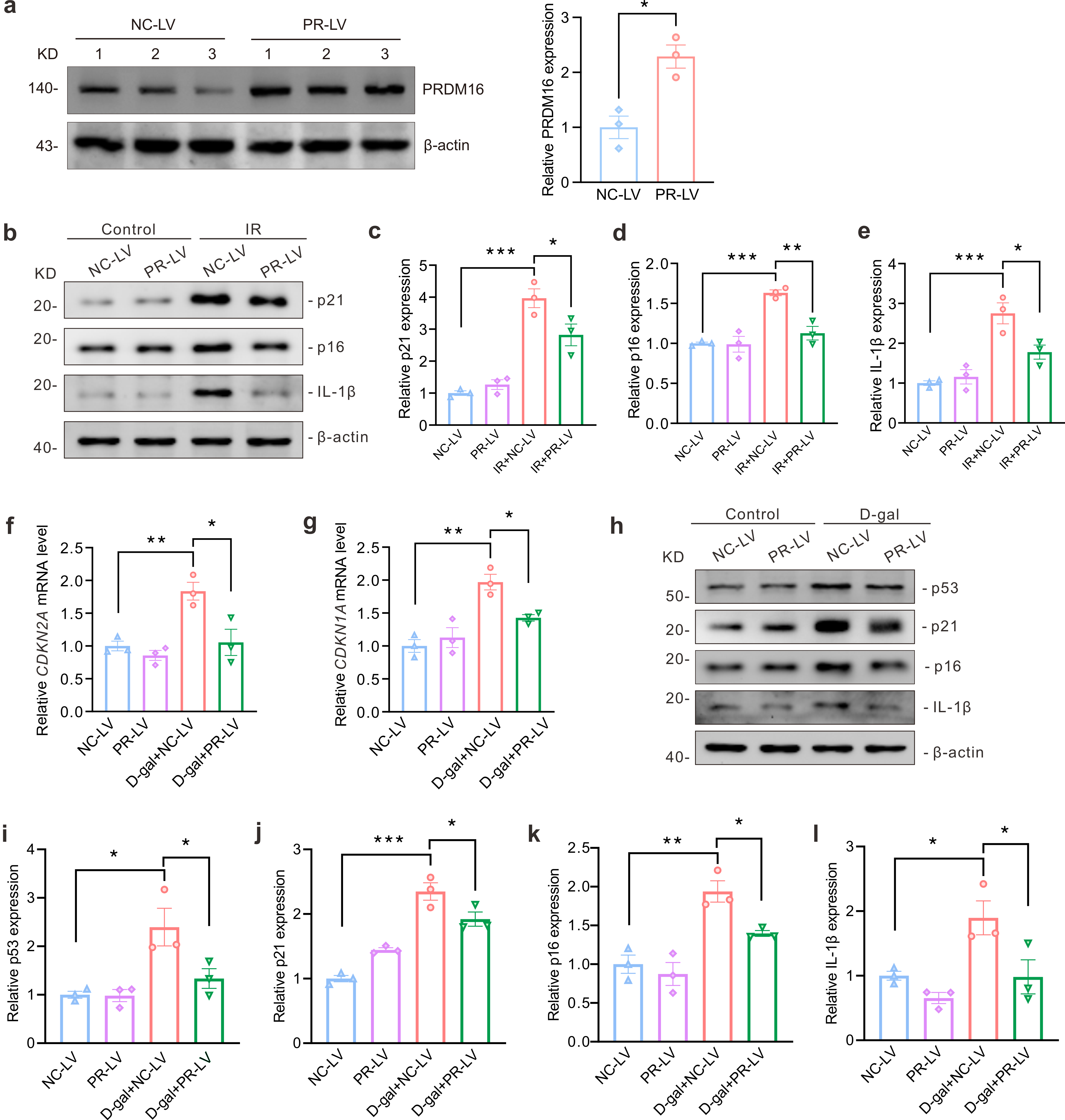


**Extended Data Figure 9. Exogenous PRDM16 alleviated senescence of HK-2 cells.**

**(a)** A representative western blot and quantification of PRDM16 in HK-2 cells transfected with negative control lentivirus (NC-LV) or PRDM16 overexpression lentivirus (PR-LV) (n=3). **(b-e)** A representative western blot **(b)** and quantification **(c-e)** of senescence and SASP markers in HK-2 cells treated with irradiation (n=3). **(f and g)** qPCR analysis of *CDKN2A* **(f)** and *CDKN1A* **(g)** in HK-2 cells treated with D-gal (n=3). **(h-l)** A representative western blot **(h)** and quantification **(i-l)** of senescence and SASP markers in HK-2 cells treated with D-gal (n=3). Data are mean ± SEM. **P* < 0.05, ***P* < 0.01 and ****P* < 0.001. Two-tailed Student’s unpaired t test analysis (a). One-way ANOVA followed by Tukey’s post-test (c-g and i-l).


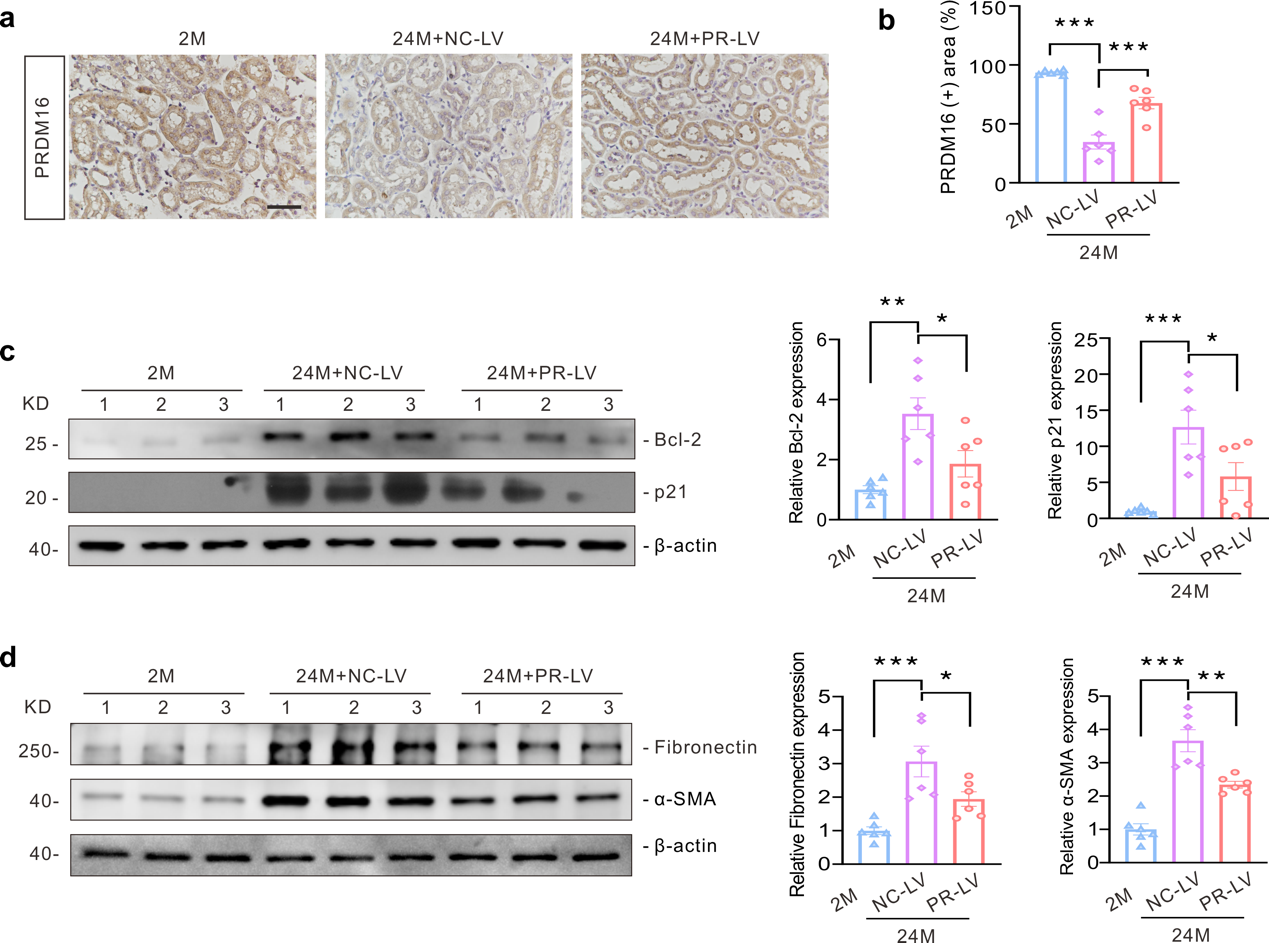


**Extended Data Figure 10. Exogenous PRDM16 attenuated kidney aging *in vivo*.**

**(a and b)** Representative IHC staining images of PRDM16 in the kidney of old mice treated with kidney lentivirus injection **(a)**. Scale bar: 50 µm. The percentage of PRDM16 positive area was calculated **(b)** (n=6). **(c)** A representative western blot and quantification of senescence markers in the kidney cortex (n=6). Numbers (1-3) represent different animals in a given group. **(d)** A representative western blot and quantification of fibrosis markers in the kidney cortex (n=6). Numbers (1-3) represent different animals in a given group. Data are mean ± SEM. **P* < 0.05, ***P* < 0.01 and ****P* < 0.001. One-way ANOVA followed by Tukey’s post-test (b-d).


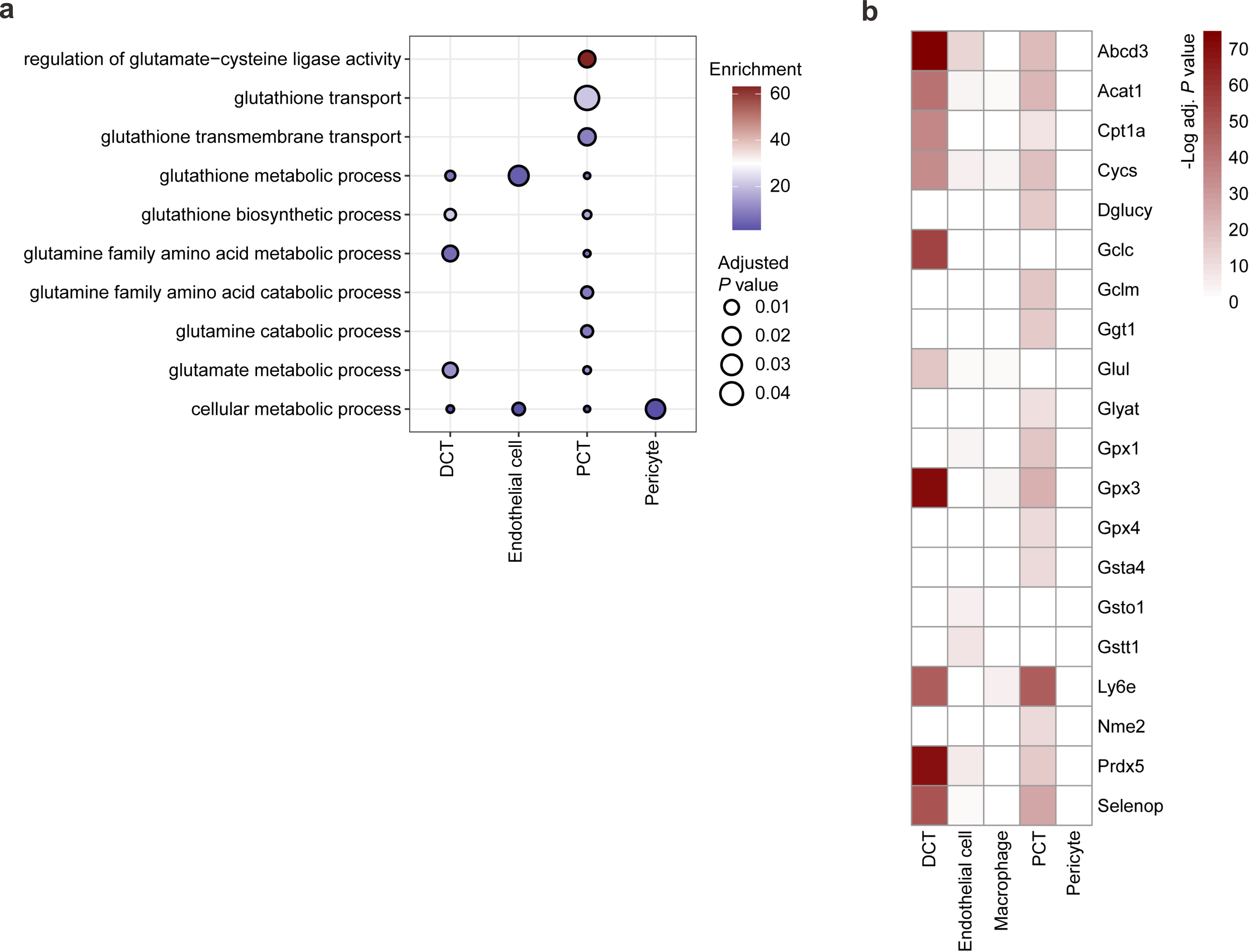


**Extended Data Figure 11. *Prdm16* deficiency affected glutathione metabolism across different cell types.**

**(a)** GO terms associated with glutathione metabolism affected by *Prdm16* deficiency across different cell types (n=3). **(b)** Glutathione metabolism related genes affected by *Prdm16* deficiency across different cell types (n=3).


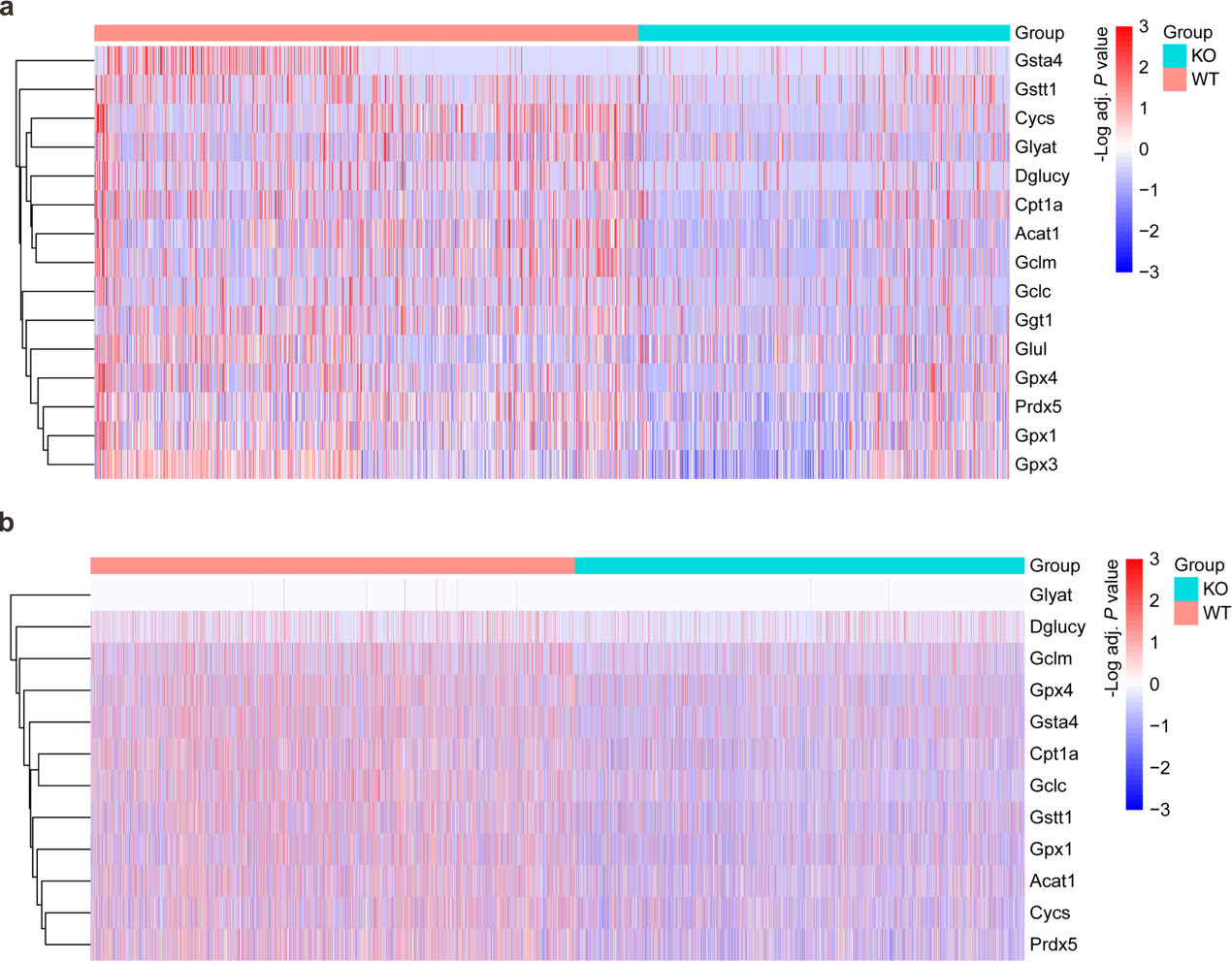


**Extended Data Figure 12. *Prdm16* deficiency downregulated the expression of glutathione metabolism associated genes in the PCT and DCT.**

**(a)** Heatmap of glutathione metabolism associated genes downregulated by PRDM16 in the PCT (n=3). **(b)** Heatmap of glutathione metabolism associated genes downregulated by PRDM16 in the DCT (n=3).


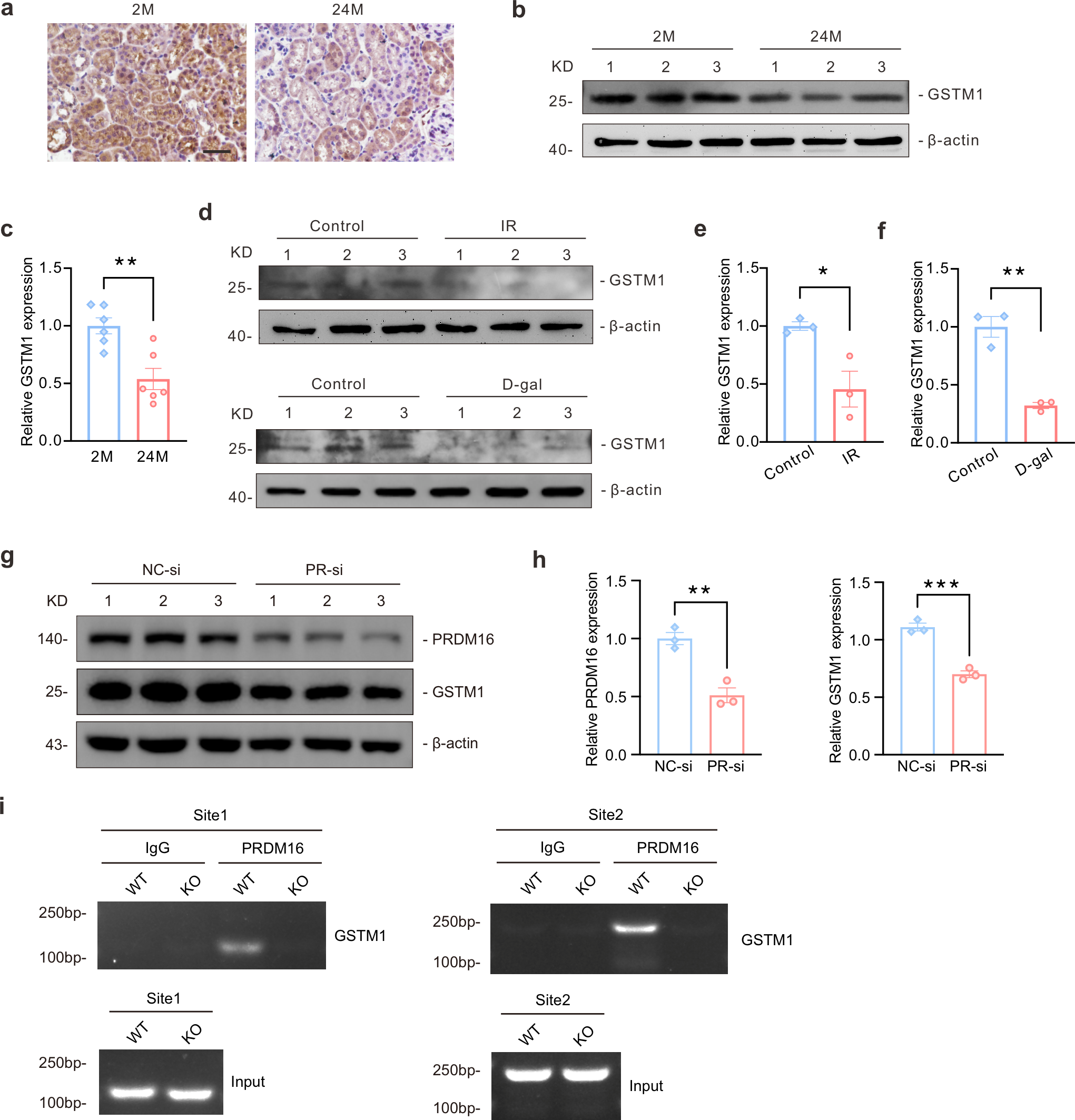


**Extended Data Figure 13. GSTM1 was downregulated in aging kidney and** **senescent HK-2 cells.**

**(a)** Representative IHC staining images of GSTM1 in the kidney of 2 months and 24 months mice. Scale bar: 50 µm. **(b and c)** A representative western blot **(b)** and quantification **(c)** of GSTM1 in the kidney cortex (n=6). Numbers (1-3) represent different animals in a given group. **(d-f)** A representative western blot **(d)** and quantification **(e and f)** of GSTM1 in irradiated or D-gal treated HK-2 cells (n=3). Numbers (1-3) represent different wells of cells in a given group. **(g and h)** A representative western blot **(g)** and quantification **(h)** of PRDM16 and GSTM1 in negative control siRNA or PRDM16 siRNA treated HEK-239T cells (n=3). Numbers (1-3) represent different wells of cells in a given group. **(i)** Representative images of ChIP-PCR conducted in renal cortex obtained from 3-week-old wide type (WT) and global *Prdm16* knockout (KO) mice using anti-PRDM16 and anti-IgG antibody. Data are mean ± SEM. **P* < 0.05, ***P* < 0.01, ****P* < 0.001. Two-tailed Student’s unpaired t test analysis (c, e, f and h).

**Extended Data Table 1. *P* value of the qPCR analysis for PRDM family members in the organs of young and aged mice.**

|  | **Kidney** | **Lung** | **Heart** | **Stomach** |
| --- | --- | --- | --- | --- |
| PRDM1 | 0.6668 | 0.5293 | 0.9394 | 0.2062 |
| PRDM2 | 0.4166 | 0.01 | 0.7869 | 0.0613 |
| PRDM4 | 0.0613 | 0.687 | 0.904 | 0.2537 |
| PRDM5 | 0.1316 | 0.1346 | 0.1332 | 0.4573 |
| PRDM6 | 0.0474 | 0.1231 | 0.2085 | 0.3943 |
| PRDM8 | 0.747 | 0.0822 | 0.4825 | 0.738 |
| PRDM10 | 0.526 | 0.0711 | 0.6012 | 0.2468 |
| PRDM11 | 0.6173 | 0.054 | 0.2491 | 0.2949 |
| PRDM12 | 0.7093 | 0.1517 | 0.4402 | 0.6105 |
| PRDM13 | 0.9487 | 0.2417 | 0.215 | 0.7631 |
| PRDM15 | 0.5331 | 0.0265 | 0.5248 | 0.2173 |
| PRDM16 | 0.0144 | 0.0279 | 0.0398 | 0.0394 |

Two-tailed Student’s unpaired t test analysis, n=3.

**Extended Data Table 2. The source of key reagents.**

| **REAGENT or RESOURCE** | **SOURCE** | **IDENTIFIER** |
| --- | --- | --- |
| **Antibodies** |  |  |
| Rabbit polyclonal anti-PRDM16 | Abcam | ab106410 |
| Rabbit polyclonal anti-p16INK4a | Abcam | ab189034 |
| Rabbit polyclonal anti-Collagen IV | Abcam | Ab6586 |
| Rabbit polyclonal anti-Bcl2 | Proteintech | 26593-1-AP |
| Rabbit polyclonal anti-P21 | Proteintech | 10355-1-AP |
| Rabbit polyclonal anti-β-actin | Proteintech | 20536-1-AP |
| Rabbit polyclonal anti-α-SMA | Proteintech | 14395-1-AP |
| Rabbit polyclonal anti-Fibronectin | Sigma | F3648 |
| Mouse monoclonal anti-gamma H2A.X | Abcam | ab26350 |
| Mouse monoclonal anti-8-oxo-dG | Sigma | MAB3560 |
| Rabbit polyclonal anti-P53 | Proteintech | 10442-1-AP |
| Rabbit polyclonal anti-p16INK4a | Proteintech | 10883-1-AP |
| Rabbit polyclonal anti-GSTM1 | Proteintech | 12412-1-AP |
| Rabbit polyclonal anti-IL1β | ABclonal | A11369 |
| Rabbit polyclonal anti-α-Tubulin | Proteintech | 11224-1-AP |
| Donkey Polyclonal Secondary Antibody to Rabbit IgG - H&L (Alexa Fluor® 594-AffiniPure) | Jackson | 711-585-152 |
| Donkey Polyclonal Secondary Antibody to Mouse IgG - H&L (Alexa Fluor® 594-AffiniPure) | Jackson | 715-585-151 |
| Donkey Polyclonal Secondary Antibody to Mouse IgG - H&L (Alexa Fluor® 488-AffiniPure) | Jackson | 715-545-150 |
| HRP Conjugated AffiniPure Goat Anti-rabbit IgG (H+L) | BOSTER | BA1055 |
| HRP Conjugated AffiniPure Goat Anti-mouse IgG (H+L) | BOSTER | BA1050 |
| Rabbit IgG | Beyotime | A7016 |
| **Chemicals** |  |  |
| Bleomycin sulfate | MCE | HY-17565 |
| Doxorubicin hydrochloride | Aladdin | D107159 |
| Lectin from Triticum vulgaris FITC Conjugate (WGA) | Sigma | L4895 |
| D-galactose | Sigma | G5388 |
| DHE Fluorescent Probe | Beyotime | S0063 |
| Isoflurane | RWD | R510-22 |
| Collagenase Type I | Gibco | 17100-017 |
| Collagenase Type II | Gibco | 17101-015 |
| Hyaluronidase | Sigma | H3757 |
| Liberase | Roche | 05401127001 |
| DNase I | Roche | 11284932001 |
| **Critical Commercial Assays** |  |  |
| Senescence β-Galactosidase Staining Kit | Beyotime | C0602 |
| GST Activity Assay Kit | Elabscience | E-BC-K278-S |
| GSH and GSSG Assay Kit | Beyotime | S0053 |
| ChIP assay kit | Beyotime | P2078 |
| DNA Purification Kit | Beyotime | D0033 |
| Dual-Luciferase Reporter Kit | Promega | E1910 |
| BD Rhapsody™ Cartridge Kit | BD | 633733 |
| BD Rhapsody™ cDNA Kit | BD | 633773 |
| BD Rhapsody™ WTA Amplification Kit | BD | 633801 |
| BD Rhapsody™ Enhanced Cartridge Reagent Kit | BD | 664887 |

**Extended Data Table 3. Primers for quantitative real-time PCR.**

| Species | Gene | Forward (5’ to 3’) | Reverse (5’ to 3’) |
| --- | --- | --- | --- |
| Mouse | *Prdm1* | AAATGGACATGGAGGACGCT | ACTCCTTACTTACCACGCCA |
| Mouse | *Prdm2* | TTTGGGGTGGATGTGCATTG | CTGCTATCTCGGGGTTGTCT |
| Mouse | *Prdm4* | AGTACCATCTCACTCGCCAC | ATGTGTGTTCCGTCCAAAGC |
| Mouse | *Prdm5* | CCGATGTGAGCTGTGCAATA | TGTGCATCTTCAGTCCGTCA |
| Mouse | *Prdm6* | CTTTTCCCAGCCGTCAGAAC | TATCGGCTTGCACTCATTGG |
| Mouse | *Prdm8* | CGGGTCGTCTCCTTACACAT | CTGTTGCTCTTTACCACCGC |
| Mouse | *Prdm10* | CCCTTGCATCCCATCCCTAA | ATCTTTCAGCTCCGATCCCC |
| Mouse | *Prdm11* | CTCACCATTCCTCAGGGCAT | TCAGCCATGAGAAGAAGCCA |
| Mouse | *Prdm12* | GGGCAGATTGAGAGTGGAGT | GGATGGAAGTGAGAGTGCCT |
| Mouse | *Prdm13* | CAACAAGCACATCCGACTCC | ACATCACTGTCCTCGCTCTT |
| Mouse | *Prdm15* | GGGGACAAGAAGTTTGCCTG | GCTTGTTCATGTTGCTCCGA |
| Mouse | *Prdm16* | CAGCACGGTGAAGCCATTC | GCGTGCATCCGCTTGTG |
| Mouse | *β-actin* | CCACCATGTACCCAGGCATT | CGGACTCATCGTACTCCTGC |
| Mouse | *Cdkn1a* | TCCAGACATTCAGAGCCACAG | AAAGTTCCACCGTTCTCGGG |
| Mouse | *Cdkn2a* | CATCTGGAGCAGCATGGAGTC | GCACCGTAGTTGAGCAGAAGA |
| Mouse | *Tp53* | TCCGAAGACTGGATGACTGC | GATCGTCCATGCAGTGAGGT |
| Mouse | *Il6* | TGCCTTCTTGGGACTGATG | ACTCTGGCTTTGTCTTTCTTGT |
| Mouse | *Il1b* | TGCCACCTTTTGACAGTGATG | ATGTGCTGCTGCGAGATTTG |
| Mouse | *Tnf* | GTAGCCCACGTCGTAGCAAA | ACAAGGTACAACCCATCGGC |
| Mouse | *Tgfb1* | CTGAACCAAGGAGACGGAATA | GGAAGGGCCGGTTCATGT |
| Mouse | *Fibronectin* | GATGAGCTTCCCCAACTGGT | CTGGGTTGTTGGTGGGATGT |
| Mouse | *Gclc* | GGGGTGACGAGGTGGAGTA | GTTGGGGTTTGTCCTCTCCC |
| Mouse | *Gclm* | CACAATGACCCGAAAGAACTGC | GAGCTGGAGTTAAGAGCCCC |
| Mouse | *Gss* | CACTGGGTCGTACCGAAGC | GTAGCCATCCCAACTCGCTC |
| Mouse | *Gstt2* | TGCCCAAGTCCACGAATACC | CCATTCTATCTCTGTTCCGTTCC |
| Mouse | *Gstm1* | TCACACAAGATCACCCAGAGC | CAATGGAACAGCCACAAAGTCA |
| Human | *LCN2* | CTGAGTGCACAGGTGCCG | TTTAGCAGACAAGGTGGGGC |
| Human | *CDKN1A* | TGGACCTGTCACTGTCTTGT | AGGCAGAAGATGTAGAGCGG |
| Human | *CDKN2A* | ACTTCAGGGGTGCCACATTC | CGACCCTGTCCCTCAAATCC |
| Human | *TP53* | GCCCCTCCTCAGCATCTTAT | AAAGCTGTTCCGTCCCAGTA |
| Human | *IL8* | GGAGAAGTTTTTGAAGAGGGCTG | ACAGACCCACACAATACATGAAG |
| Human | *IL1B* | TTCGAGGCACAAGGCACAA | AGTCATCCTCATTGCCACTGTAA |
| Human | *TNF* | TCTCCTTCCTGATCGTGGCA | CAGCTTGAGGGTTTGCTACAAC |
| Human | *KL* | CTGTGACTTTGCTTGGGGAG | AGTCCAGGGAGAAGCGAAAA |
| Human | *β-actin* | CTCACCATGGATGATGATATCGC | AGGAATCCTTCTGACCCATGC |
| Human | *GSTM1* | TCTGCCCTACTTGATTGATGGG | TCCACACGAATCTTCTCCTCT |
| Human | ChIP primer1 | AAGCCCTGGGAGTAGCTTTC | TGAGACACCAGCCAGATCCT |
| Human | ChIP primer2 | CTTACAGTGACAGGGGCTGA | ACCAGCCAGATCCTAACGGA |
| Human | ChIP primer3 | CTCTTACAGTGACAGGGGCTG | TGAGACACCAGCCAGATCCTA |
| Human | ChIP primer4 | GCAGTCAACCCCAGGCTAAT | GTATGAGACGGTGGCTCCAG |
| Human | ChIP primer5 | TAAAGCAGTCAACCCCAGGC | CAGTATGAGACGGTGGCTCC |
| Human | ChIP primer6 | CAGTCAACCCCAGGCTAATCC | GGCCTTTGAAGTCACACCGT |
| Mouse | ChIP  site1 | GGCCCCATGACAATCACCAG | GTGGTCATCTTCCACAAGCCA |
| Mouse | ChIP  site2 | AGGAATGATGAGGCTGGTGAG | TCAATTCAGCTTCCCCAGAGAG |
| Rat | *Prdm16* | GAAGGTGTGTTGAGGAGCGA | GCATCATTGCGTATGCCTGG |
| Rat | *β-actin* | ATCATTGCTCCTCCTGAGCG | GAAAGGGTGTAAAACGCAGCTC |

**Extended Data Ethical Approval**


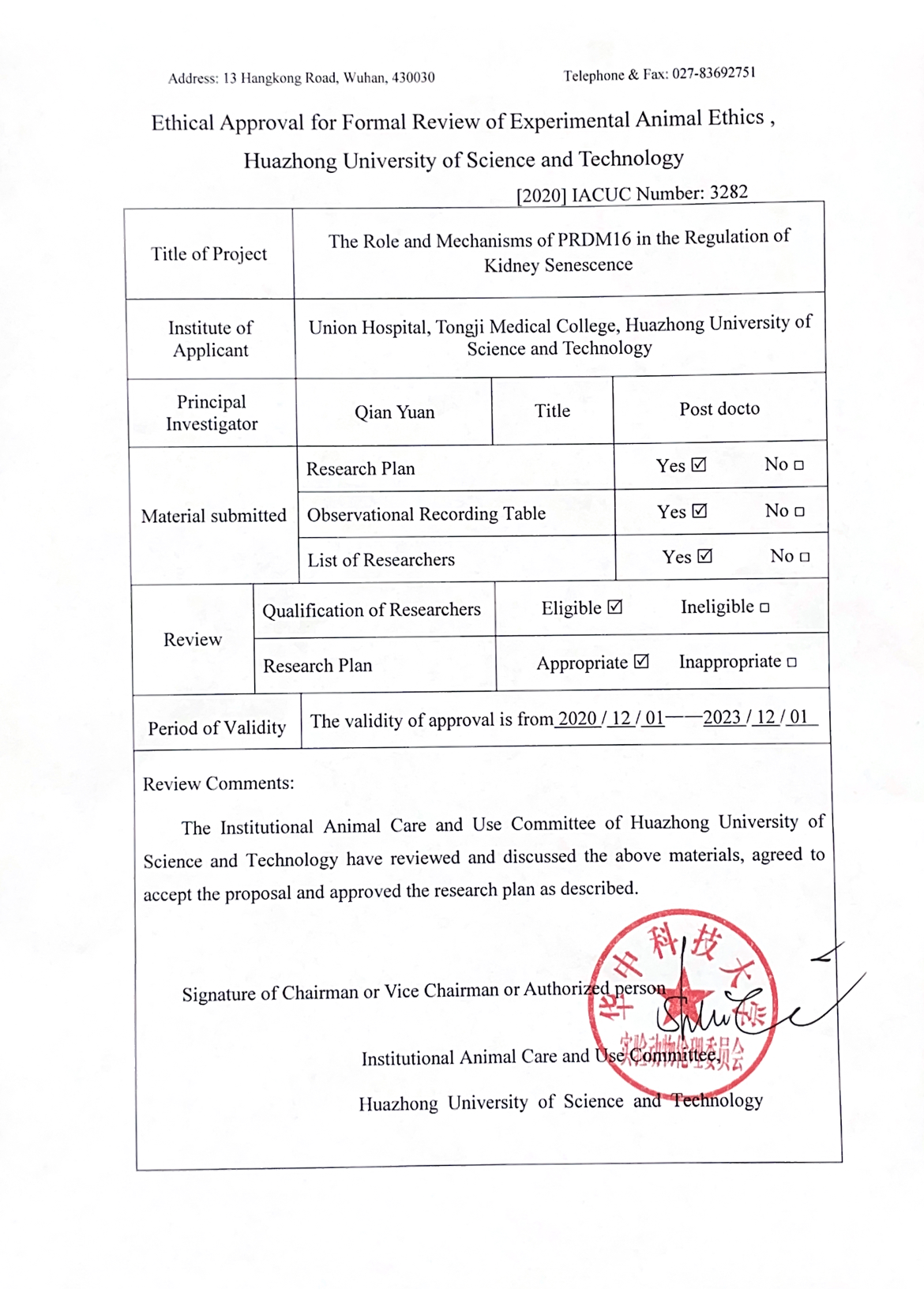

Supplement: Supplementary file 1 — Supporting Information [file ADVS-12-e01233-s001.docx]
